# A systematic review and meta‐ethnographic synthesis of Mindfulness‐based Cognitive Therapy for people with major depression

**DOI:** 10.1002/cpp.2773

**Published:** 2022-08-15

**Authors:** Kate Williams, Samantha Hartley, Susanne Langer, Mizla Manandhar‐Richardson, Melissa Sinha, Peter Taylor

**Affiliations:** ^1^ Division of Psychology & Mental Health, School of Health Sciences University of Manchester, Manchester Academic Health Sciences Centre Manchester UK; ^2^ Greater Manchester Mental Health NHS Foundation Trust Manchester UK; ^3^ Department of Psychology, Faculty of Health, Psychology, and Social Care Manchester Metropolitan University Manchester UK; ^4^ Pennine Care NHS Foundation Trust Ashton‐under‐Lyne UK; ^5^ Cheshire and Wirral Partnership NHS Foundation Trust Chester UK

**Keywords:** major depression, MBCT, meta‐ethnography, meta‐synthesis, mindfulness‐based cognitive therapy, qualitative

## Abstract

**Background:**

Mindfulness‐based Cognitive Therapy (MBCT) is a relapse‐prevention intervention for people experiencing major depression. Three qualitative meta‐syntheses investigating experiences of taking part in MBCT and/or Mindfulness‐based Stress Reduction (MBSR) across different diagnostic populations reported themes including control, choice, group processes, relationships and struggles. As multiple studies have been published since, we aimed to update, systematically review and synthesize the experiences of participants with depression taking part in MBCT.

**Methods:**

Four databases were searched systematically (PsycInfo, Web of Science, Medline and CINAHL) up to and including 12 November 2021. Twenty‐one qualitative studies met the review criteria. All papers were rated as fair using a quality appraisal tool. Meta‐ethnography was applied.

**Results:**

Across 21 studies of participants with current or previous depression who had participated in MBCT, three overarching themes were developed: ‘Becoming skilled and taking action’, ‘Acceptance’ and ‘Ambivalence and Variability’. Participants became skilled through engagement in mindfulness practices, reporting increased awareness, perspective and agency over their experiences. Participants developed acceptance towards their experiences, self and others. There was variability and ambivalence regarding participants' expectations and difficulties within mindfulness practices.

**Limitations:**

Many studies were conducted in MBCT‐research centres that may hold conflicts of interest. Many studies did not address the impact of the participant–researcher relationship thus potentially affecting their interpretations. Studies were skewed towards the experiences of female participants.

**Conclusions:**

Our findings help to enhance participant confidence in MBCT, alongside understanding the processes of change and the potential for difficulties. MBCT is beneficial and provides meaningful change for many but remains challenging for some.

**Key Practitioner Message**

- This meta‐ethnography of 21 qualitative studies generated three overarching themes.
- MBCT enables the development of new skills and different ways of responding, as well as a shift towards accepting experiences, self and others.
- Meaningful change is variable across participants and there is the potential for difficult experiences to arise both during and beyond MBCT.
- This study gives practitioners a greater understanding of the processes of change, alongside the benefits and challenges of MBCT.
- We highlight clear implications and include recommendations for practice for MBCT teachers, clinicians and researchers.

## 1 INTRODUCTION

Major depression affects approximately 264 million people worldwide and has an estimated lifetime prevalence of 10.8% (James et al., 2018; Lim et al., 2018). Individuals can experience multiple episodes of depression with the risk of further depression increasing to 90% for people with three or more past episodes (Kessing et al., 2004; Kupfer, 1991). The cognitive vulnerability theory of depression proposed that relapse into depression is maintained by each episode subsequently embedding the associations between low mood and negative thinking (Lau et al., 2004; Segal et al., 2013). As a result, Mindfulness‐based Cognitive Therapy (MBCT; Segal et al., 2013) was developed by incorporating Mindfulness‐based Stress Reduction (MBSR), which includes an experiential understanding of mindfulness‐based meditation (Kabat‐Zinn, 1990), with elements of cognitive therapy specific to depression. MBCT focuses on developing awareness of triggers to depression and adoption of a kinder, non‐judgemental attitude to internal experiences (Segal et al., 2013). MBCT is recommended by the National Institute for Health and Care Excellence (NICE; National Institute for Health and Care Excellence, 2022) as a relapse‐prevention intervention for recurrent and less severe current depression. Two meta‐analyses of 14 and 9 randomised controlled trials (RCTs) have shown that MBCT is effective in preventing relapse when compared to treatment as usual (TAU; e.g., maintenance antidepressants, usual support) or active control (e.g., Cognitive Behavioural Therapy, CBT) groups (Kuyken et al., 2016; McCartney et al., 2021).

Simultaneously, a number of qualitative studies have attempted to explore the experiences of MBCT for individuals with depression. Three qualitative meta‐syntheses have synthesized these studies: two studies focused on both MBCT and MBSR (Malpass et al., 2012; Wyatt et al., 2014) and a third study focused only on MBCT but across diagnostic groups additional to depression (Cairns & Murray, 2013). In the two meta‐syntheses exploring both MBCT and MBSR, a number of themes were reported around participants' experiences. While it is not possible to describe all the themes in sufficient detail here, overall the themes tended to capture participants' experiences of having become more aware of maladaptive coping strategies and moving towards learning new skills, benefits and difficulties within group processes, improved relationships, increases in personal control, choice, alongside ongoing struggles with mindfulness (Malpass et al., 2012; Wyatt et al., 2014). Specific to MBCT, Cairns and Murray (2013) reported similar themes as well as describing that participants noticed increased feelings of acceptance around their experiences (e.g., thoughts and feelings), the role of expectations on overall outcome and changing feelings towards the self. However, these systematic reviews either focused on different MBIs, thus were not specific to MBCT, or explored the experiences of MBIs across a range of clinical presentations. Given that MBCT was designed specifically to target relapse prevention in individuals currently in remission from depression, none of the three reviews allowed specifically for a more in‐depth focus on the experiences of MBCT for remitted depression.

However, over the last few years, 15 additional qualitative studies specifically exploring MBCT for depression have been published. This is a notable increase, which warrants not only an update to the findings but also a more specific understanding and synthesis of MBCT for people with experiences of depression. Consequently, due to this focus on participants' qualitative experiences of MBCT for depression, we elected not to include studies focused on MBSR, in order to retain the focus of the review. A meta‐ethnographic approach was applied (Noblit & Hare, 1988). A meta‐ethnography moves beyond the individual ‘parts’ of studies towards a ‘greater whole’, allowing for novel interpretations, instead of solely an aggregation of study findings (Noblit & Hare, 1988). We applied the seven steps of meta‐ethnography (Noblit & Hare, 1988) in order to synthesize the results from studies specifically exploring individual experiences of MBCT for depression. Our aim for this meta‐ethnography was to better understand the therapeutic processes, benefits and challenges of MBCT specifically for people with experiences of depression, moving beyond prior systematic reviews, which have been limited in their specific understanding of MBCT for depression. From a clinical perspective, it will be important to elucidate the processes involved in how people experience MBCT and therefore how MBCT works to prevent relapse into depression. Therefore, our primary research question was ‘How do participants with depression experience the therapeutic processes within MBCT?’ with a focus on what was helpful alongside the challenges of MBCT.

## 2 METHOD

### 2.1 Protocol pre‐registration

The review protocol was pre‐registered on the International Prospective Register of Systematic Reviews (PROSPERO; registration number CRD42020170979). This review was conducted in line with both the updated ‘Preferred Reporting Items for Systematic Reviews and Meta‐analyses’ (PRISMA; Page et al., 2021) and the ‘Improving Reporting of Meta‐Ethnography’ (eMERGe reporting guidance; France et al., 2019) guidelines. Tables S1–S3 include the completed checklists.

### 2.2 Inclusion and exclusion criteria

#### 2.2.1 Inclusion criteria

The intervention must have been MBCT (i.e., not MBSR or another mindfulness‐based intervention [MBI]), delivered in a group or one‐to‐one setting, either face‐to‐face and/or via remote methods (e.g., online). The recipients of MBCT could include individuals, couples, or families, provided that all of the remaining inclusion and exclusion criteria were met. Minor amendments to MBCT were acceptable provided they did not deviate from the focus on relapse‐prevention in depression or extensively from the manual (Segal et al., 2013). Participants must have either experienced symptoms in line with major depression (e.g., meeting diagnostic criteria set out by the DSM‐IV/V or ICD‐10), or were currently self‐reporting or had previously experienced self‐reported symptoms of depression. Both adults and adolescents could be included, provided that adolescents were aged over 12 years to reduce significant MBCT adaptations and to ensure participants could adequately self‐report on their experiences (both studies with adolescents included age ranges with a minimum age of 12; see Section 3). Studies were either peer‐reviewed or unpublished publications and had to have employed qualitative or mixed methodologies (e.g., studies combining both quantitative and qualitative methods); for the latter, only the qualitative data was analysed. All studies were required to be written in English.

#### 2.2.2 Exclusion criteria

Studies reporting solely on quantitative methods, systematic reviews, meta‐analyses, commentaries, narrative reviews, protocols and case studies were excluded. Studies of other psychological therapies or studies including Acceptance and Commitment Therapy (ACT; Hayes et al., 1999), Dialectical Behaviour Therapy (DBT; Linehan, 1993) or Yoga were excluded. Although the latter interventions contained elements of mindfulness, they constituted significant deviations from the manualized MBCT. Participants in the individual studies should not have experienced symptoms in line with or been diagnosed with other non‐unipolar depression DSM‐IV/V or ICD‐10 diagnoses such as bipolar disorder or psychosis, given that the content and focus of MBCT would have been changed significantly to meet the needs of the participants.

### 2.3 Search strategy

The search was conducted across four databases: PsycInfo, Web of Science, Medline and CINAHL. The following search terms were used to search titles and abstracts: (mindfulness* or mindfulness‐based* or MBCT) and (depress*) and (interview* or focus group* or case stud* or experience* or qualitative or mixed method*). MeSH terms were selected in PsycInfo and Medline (‘mindfulness‐based interventions’, ‘mindfulness’, ‘major depression’, ‘qualitative methods’ and ‘mixed methods research’) with ‘suggested subject terms’ selected instead on Web of Science and CINAHL (e.g., ‘mindfulness’, ‘depression’ and ‘treatment outcomes’). There were no prior date limits to enable identification of all relevant papers published up until the date of the search. The search was initially conducted on 2 October 2020, with an updated search completed on 12 November 2021. The updated search followed the same methodology and analysis as highlighted here.

Duplicate publications were manually removed. Titles and abstracts were screened by the lead researcher to determine eligibility. Studies not meeting the inclusion criteria at this stage were excluded. All remaining studies were taken forward for full text screening to determine eligibility. To check for additional papers not detected by the search, we checked reference lists from the included studies, three reviews (Cairns & Murray, 2013; Malpass et al., 2012; Wyatt et al., 2014), and forward citation searching. All study authors (*N* = 21) were emailed to enquire about any further publications, either unpublished or in preparation; all 21 authors replied but no eligible studies were identified. Four authors were contacted for further clarification over study methods (Table S4). Fifteen percent of papers were screened in parallel by an independent researcher, and independently of each other. Agreement over study inclusion was determined according to the eligibility criteria noted above and through reporting and discussion reasons for exclusion of papers, in line with guidelines from Tawfik et al., 2019. Using this method, parallel screening resulted in 95.45% agreement. Discrepancies around the inclusion of one paper (Bailie et al., 2012) concerned queries around the sample and criteria for eligibility. Both researchers discussed this and agreed that this study was eligible for inclusion as it focused on parents with histories of recurrent depression who had participated in MBCT. Both researchers came to 100% agreement over the final inclusion of the 21 studies.

### 2.4 Quality appraisal

The Critical Appraisal Skills Programme (CASP, 2018) was used for quality appraisal, due to its consideration of reflexivity in qualitative studies. The CASP was not used to exclude or categorize studies. Ratings were carried out by the lead researcher and two of the co‐authors (MM; MS), with discrepancies discussed and resolved as required. In an effort to avoid bias, only MM and MS conducted the quality appraisal for the final paper (Williams et al., 2021) as this paper was conducted by the lead author (KW); neither MM or MS were involved in that publication.

### 2.5 Analysis

We followed the seven steps of meta‐ethnography (Noblit & Hare, 1988; Table S5) alongside additional, updated methodology guidance from Sattar et al. (2021), France et al. (2019) and Toye et al. (2014). The seven steps were conducted primarily by the lead researcher (KW) alongside regular discussion and review of key concepts, themes and decisions at each of the seven steps. Of note, in an effort to reduce bias, the themes for the final included paper (Williams et al., 2021) were reviewed by a co‐author (MM) as this was the lead researcher's own publication. The studies were uploaded into the qualitative analysis software package NVivo (QSR International, 1999), and the results and discussion sections were coded verbatim, using the original authors' own words with minor paraphrasing. To become immersed in the data, the lead researcher read the studies multiple times noting down key concepts and themes (Cahill et al., 2018; Lee et al., 2015). Only second‐order constructs (study author's themes, subthemes and interpretations) were analysed as first‐order quotes have been purposely selected by the study authors and may not sufficiently represent all participants (Atkins et al., 2008; France et al., 2019; Lee et al., 2015). Indeed, Noblit and Hare (1988) advised that meta‐ethnographies should not involve analysis of raw participant data, but instead analyse the interpretations from study authors. However, as a sensitivity analysis, the first‐order constructs are in Tables S6 and S7; there were no major differences between the first‐ and second‐order constructs. At the early stages of analysis, key themes, concepts and interpretations were coded in batches; two studies with methodological concerns (both dissertation theses) and two studies which included younger populations were initially coded separately to assess how they differed to the remaining studies. There were no substantive differences as the majority of codes appeared within and across the batches. Therefore, all codes were combined in further steps of the analysis process.

Second‐order constructs were condensed into categories through identifying the similarities and differences across the studies (Table S8). In order to preserve the context and meaning of the relationships between the second‐order constructs within and across studies, we conducted a reciprocal translation (Noblit & Hare, 1988). Using a reciprocal translation, categories were directly compared and condensed into second‐order constructs through constructing narrative comparisons and visual maps (Melendez‐Torres et al., 2015). Specifically, narrative comparisons and visual thematic maps were developed to detail the relationships between the codes, metaphors, quotes and early themes. This allowed for visualizing the similarities and differences between the categories, with a view to comparing and condensing the categories to subsequently develop these into themes. This process enabled us to identify similarities between the categories (e.g., some categories were visually mapped out and subsequently combined into one second‐order construct due to their similarities across the included studies). Similar translations were clustered together and we used a line of argument synthesis to develop third‐order constructs (new interpretations of the relationships between the second‐order constructs; Toye et al., 2014; Noblit & Hare, 1988). Hypothesized interactions between the higher‐order themes were developed based on the accounts of the participants. This approach enabled a move beyond aggregating studies towards developing new interpretations and new conceptual ways of understanding the studies (Sattar et al., 2021).

### 2.6 Reflexivity

A critical realist approach was adopted during the analysis process, whereby an ultimate reality exists whilst acknowledging that this reality is shaped by the researcher's and participants' assumptions, experiences, culture, social and political experiences. The lead researcher was aware that they held an interest in, knowledge of, and experience with facilitating MBCT groups and researching MBCT for depression. In line with a critical realist approach, they maintained a reflective log to document expectations, assumptions and interpretations throughout analysis. The lead researcher engaged in supervision and regular discussion of the themes with researchers who do not have experience with MBCT thus helping to reflect on their active role within the analysis process. Further detail regarding reflexivity can be found in our reflexive statement in Supporting Information S7.

## 3 RESULTS

### 3.1 Screening

**FIGURE 1:**
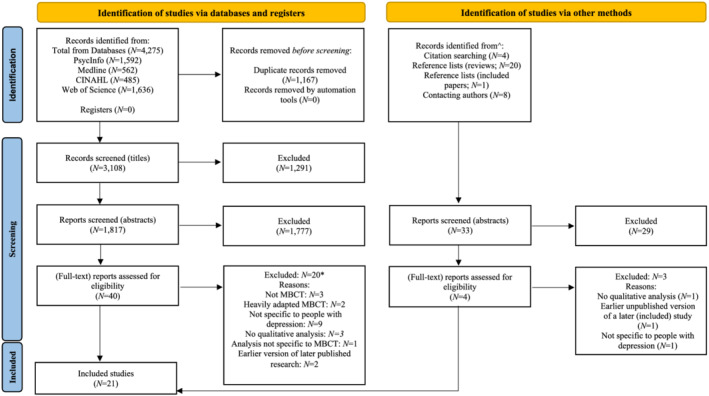
Preferred Reporting Items for Systematic Reviews and Meta‐analyses (PRISMA) 2020 flow diagram (Page et al., 2021). *Note:* ^dates these searches were conducted are in Table S4; *full reasons are in Supporting Information S7; two studies were identified as eligible in the updated search on 12 November 2021.

Twenty‐one studies were included in the synthesis. Figure 1 details the search process.

### 3.2 Study characteristics

Study characteristics are in Table 1. Most studies were conducted in the UK (*N* = 14), with others across mainland Europe (*N* = 3) or the USA (*N* = 4). Nineteen studies included adult samples; two studies included adolescent samples. Only 15 studies reported on gender which was skewed towards female (range 63%–100%). The majority of studies reported on group‐based, face‐to‐face delivery of MBCT (*N* = 20) with one study delivering online MBCT. Most studies (*N* = 15) included participants with histories of multiple ‘episodes’ of depression but were either currently free from symptoms (‘full remission’) or experiencing mild symptoms (‘partial remission’). The remaining studies (*N* = 6) included participants who were currently experiencing symptoms in line with ‘mild’ to ‘moderate’ severities. Interviews were conducted either post‐MBCT (*N* = 5; one of which included pre‐MBCT), during follow‐up (*N* = 6) or at both post‐MBCT and follow‐up (*N* = 6, one of which included pre‐MBCT). Semi‐structured or open interviews were administered (*N* = 18); the remaining studies used written questionnaires (*N* = 1) or a mix of interviews and questionnaires (*N* = 2). All studies employed qualitative analysis.

**TABLE 1:** Study characteristics

Authors	Year country	*N*	Age Age range Gender	MBCT delivery	Depression	Time of interview	Data collection	Analysis	Aims/focus
Mason and Hargreaves^a^	2001 UK	7	*M* = 43.14 SD = 12.12 24–59 71% female	Group Face: Face 8 weeks	Full/partial remission	*N* = 4 post‐MBCT *N* = 3: 12–30 months post‐MBCT	Open interviews (*N* = 4) which guided interviews for *N* = 3	Grounded theory	Therapeutic processes involved in MBCT
Ma^a^ ^,^ ^b^	2002 UK	35	*M* = 42.89 SD = 8.39 ‐ 73% female	Group Face:Face 8 weeks	Full/partial remission	12 months post‐MBCT	Semi‐structured interviews Mixed methods thesis	IPA	To understand MBCT in preventing relapse
Finucane and Mercer	2006 UK	11	*M* = 43.08 SD = 9.38 29–58 77% female	Group Face:Face 8 weeks	Current symptoms of depression.	3 months post‐MBCT	Semi‐structured interviews Mixed methods	Framework approach, including thematic matrices	Acceptability and feasibility of MBCT in primary care
Smith et al.	2007 UK	30	*M* = 70.5 ‐ 65–88 63% female	Group Face:Face 8 weeks	Full/partial remission	2 weeks post‐MBCT After 12–13 months	Interviews Mixed methods	Thematic analysis	What MBCT characteristics were helpful/not helpful
Allen et al.^a^	2009 UK	20	*M* = 51.45 SD = 9.51 ‐ 85% female	Group Face:Face 8 weeks	Full/partial remission	12 months post‐MBCT	Semi‐structured interviews. (RCT)	Thematic analysis	Experiences of MBCT
Cebolla and Barrachina^b^	2009 Spain	32	‐*(adults)*	Group Face:Face 8 weeks^d^	Current depression and/or anxiety	Post‐MBCT 3 months follow‐up	Written questionnaires	Content analysis	Experiences of MBCT
Baillie et al.^a^ ^,^ ^c^	2012 UK	16	‐ *(adults)*	Group Face:Face 8 weeks	Full/partial remission	12 months post‐MBCT	Interviews Embedded in an RCT	Thematic analysis	Parents with recurrent depression and relationships with their children
Hopkins and Kuyken^a^	2012 UK	13	*M* = 48.92 SD = 12.22 23–67 69% female	Group Face:Face 8 weeks	Full/partial remission	Within 4 years post‐MBCT	Semi‐structured interviews	IPA	Benefits and barriers to attending reunions
Worsfold	2013 Denmark	15	‐ *(adults)* 93% female	Group Face:Face 8 weeks	Full/partial remission	Pre‐ and post‐MBCT	Semi‐structured interviews	Phenomenological approach	Body awareness and MBCT
Ames et al.	2014 UK	7	‐ 12–18 100% female	Group Face:Face 8 weeks	Symptoms of depression, with/without anxiety	Post‐MBCT 1 month follow‐up	Semi‐structured interviews Mixed methods	IPA	MBCT for adolescents
Bihari and Mullan^a^	2014 UK	11	‐ *(adults)*	Group Face:Face 8 weeks	Full/partial remission	MBCT within the last 3 years	Semi‐structured interviews	Grounded theory	Interpersonal change processes
Boggs et al.^a^	2014 USA	38	*M* = 46.89 SD = 12.38 71.1% female	Group web‐based (MMB)	Full/partial remission	Exit interview after MMB	Interviews Mixed methods	Content analysis, grounded theory	Experiences of MBCT
Lilja et al.	2015 Sweden	19	‐ 30–68 ‐	Group Face:Face 8 weeks	Full remission	12 months post‐MBCT	Semi‐structured interviews	Thematic analysis	How primary care participants perceive MBCT
Murphy and Lahtinen	2015 UK	6	‐ 41–60 83% female	Group Face:Face 8 weeks	Full/partial remission	Between 3 and 12 months post‐MBCT	Semi‐structured interviews	IPA	Relationship with thoughts in MBCT
Di Toro	2017 USA	11	*M* = 52.45 ‐ 31–70 73% female	Group Face:Face 8 weeks	Mild to moderate symptoms	Post‐MBCT	Open‐ended interviews Mixed methods thesis	Thematic analysis	Feasibility of MBCT in primary care
Chesin et al.	2018 USA	15^5^ 4^6^	*M* = 41.6 SD = 14.4 ‐ 80% female	Group Face:Face 9 weeks	Full/partial remission Current suicidal ideation	3 weeks post‐MBCT^e^ 1 week post‐MBCT^f^	Online surveys^e^ Focus groups^f^	Thematic analysis	MBCT for people at high risk of suicide.
Racey et al.^a^ ^,^ ^g^	2018 UK	7	‐ 14–18 Unclear gender split	Group Face:Face 8 weeks^d^	Full/partial remission	Post‐MBCT	Semi‐structured interviews. Mixed methods	Thematic analysis	Feasibility and acceptability for adolescents
Williams et al.	2018 UK	13	*M* = 71.25 SD = 4.07 65–78 69% female	Group Face:Face 8 weeks	Full/partial remission	Pre‐ and post‐MBCT 6 months post‐MBCT	Semi‐structured interviews	Thematic analysis	Reflections on MBCT
Tickell et al.^a^ ^,^ ^b^	2020 UK	42	*M* = 51.88 SD = 10.51 25–72 74% female	Group Face:Face 8 weeks	Full/partial remission	Post‐MBCT 2 years follow‐up	Written post‐MBCT Semi‐structured interviews follow‐up (RCT)	Thematic analysis	Experiences of MBCT and AD to support recovery
Canby et al.^a^ ^,^ ^b^	2021 USA	88	‐ 18–65 Unclear in context of qualitative sample	Group Face:Face 8 weeks	Current mild to severe depression and anxiety^h^	3 months post‐MBCT	Structured interviews Mixed methods (RCT)	Content analysis using codebook categories	How instructor and group factors impacted therapeutic journeys post‐MBCT
Williams et al.	2021 UK	35	*M* = 37.6 SD = 10.99 ‐ 74% female	Group Face:Face 8 weeks	Full/partial remission	Post‐MBCT 3, 6, and 12 months post‐MBCT^e^	Semi‐structured interviews	Reflexive thematic analysis	Exploring benefits and challenges of MBCT

*Note*: ‘‐’ = data not given/able to be calculated; IPA = Interpretive Phenomenological Analysis; (RCT) = (took place within a) Randomised Controlled Trial; MMB = Mindful Mood Balance; AD = antidepressants; MDD = Major Depressive Disorder; GAD = Generalised Anxiety Disorder.

^a^ Conducted in centres with MBCT expertise.

^b^ Authors contacted further.

^c^ Participants were parents for at least one child.

^d^ Minor content adjustments to suit population.

^e^ Online surveys.

^f^ Focus groups.

^g^ Only data from young people is included.

^h^ Additional inclusion/exclusion information provided by Canby et al.: all participants met criteria of mild–severe levels of depression (scores of 10–48 on the inventory of depressive symptomology) and persistently high levels of negative affect (>18 on PANAS negative affect scale).

One study also included interviews with parents and staff; only the findings from interviews with adolescents were included. Although parents participated in MBCT, this was to better support their child through depression (see Table S9). While most studies aimed to explore therapeutic processes of MBCT, two studies focused specifically on exploring the relationship with the body (Worsfold, 2013) or thoughts (Murphy & Lahtinen, 2015). Finally, 10 studies were conducted in Universities connected with extensive clinical and research expertise in MBCT, which may have enriched the findings but may have led to unintended assumptions and expectations impacting on their interpretations. We conducted sensitivity analyses based on some of the study characteristics in order to explore whether there were any noticeable differences between the overarching themes and second‐order constructs, based on the country of origin (UK/Europe vs. USA), method of MBCT delivery (face: face vs. online) and depression status (full/partial remission vs. current symptoms). There were some minor differences between themes within and across studies which are discussed throughout the manuscript and displayed in Tables S6 and S7.

### 3.3 Quality appraisal

The quality of the studies was rated as fair, with 8 of the 21 studies rated as lower quality overall (Table 2). Only 12 studies sufficiently justified their choice of qualitative design and analysis. Thirteen studies lacked information regarding the participant‐researcher relationship; therefore, it is difficult to assess the impact this had on the researcher's interpretations in the original studies. Full details are in Table S10.

**TABLE 2:** CASP quality appraisal

Study^a^	1. Aims stated	2. Appropriate for qualitative methods	3. Appropriate research design	4. Appropriate recruitment strategy	5. Appropriate data collection	6. Participant researcher relationship	7. Ethical issues	8. Rigorous analysis	9. Statement of findings	10. Value
1	✓	✓	✓	?	✓	✓	✗	✓	✓	?
2	✓	✓	✓	✓	✓	✗	✓	✓	?	?
3	✓	✓	?	✓	✓	?	✓	✓	✓	✓
4	✓	✓	✓	✓	?	?	?	✓	✓	✓
5	✓	✓	✓	✓	✓	?	✓	✓	✓	✓
6	✗	✓	?	✗	✓	✗	✗	✗	?	✗
7	✓	✓	✓	✓	✓	✓	?	✓	✓	✓
8	✓	✓	✓	✓	✓	✓	✓	✓	✓	✓
9	✓	✓	?	✓	✓	✗	?	?	?	✓
10	✓	✓	?	?	?	?	✓	✗	✓	✓
11	✓	✓	✓	✓	✓	✓	✓	✓	✓	✓
12	✓	✓	?	?	✓	?	✓	✓	✓	✓
13	✓	✓	✓	✓	✓	✓	✓	✓	✓	✓
14	✓	✓	✓	✓	✓	✓	✓	✓	✓	✓
15	✓	✓	✗	✓	✓	?	✓	✓	✓	✓
16	✓	✓	?	✓	✓	✗	✓	✓	✓	✓
17	✓	✓	✗	✓	✓	✗	✓	?	?	✓
18	✓	✓	✓	✓	✓	?	✓	✓	✓	✓
19	✓	✓	?	✓	✓	✓	✓	✓	✓	✓
20	✓	✓	✓	?	✓	?	✓	?	✓	✓
21	✓	✓	✓	✓	✓	✓	✓	✓	✓	✓

*Note*: Individual item ratings: ✓ = ‘Yes’; ✗=‘No’; ? = ‘Cannot tell’. The quality appraisal for paper number 21 was not carried out by the lead author as they were the lead author on this paper but instead carried out by two of the co‐authors (MM and MS).

^a^ Study: 1 = Mason and Hargreaves; 2 = Ma; 3 = Finucane and Mercer; 4 = Smith et al.; 5 = Allen et al.; 6 = Cebolla and Barrachina; 7 = Baillie et al.; 8 = Hopkins and Kuyken; 9 = Worsfold; 10 = Ames et al.; 11 = Bihari and Mullan; 12 = Boggs et al.; 13 = Lilja et al.; 14 = Murphy and Lahtinen; 15 = Di Toro; 16 = Chesin et al.; 17 = Racey et al.; 18 = Williams et al.; 19 = Tickell et al.; 20 = Canby et al.; 21 = Williams et al.

### 3.4 Translation and synthesis of second‐order constructs

**FIGURE 2:**
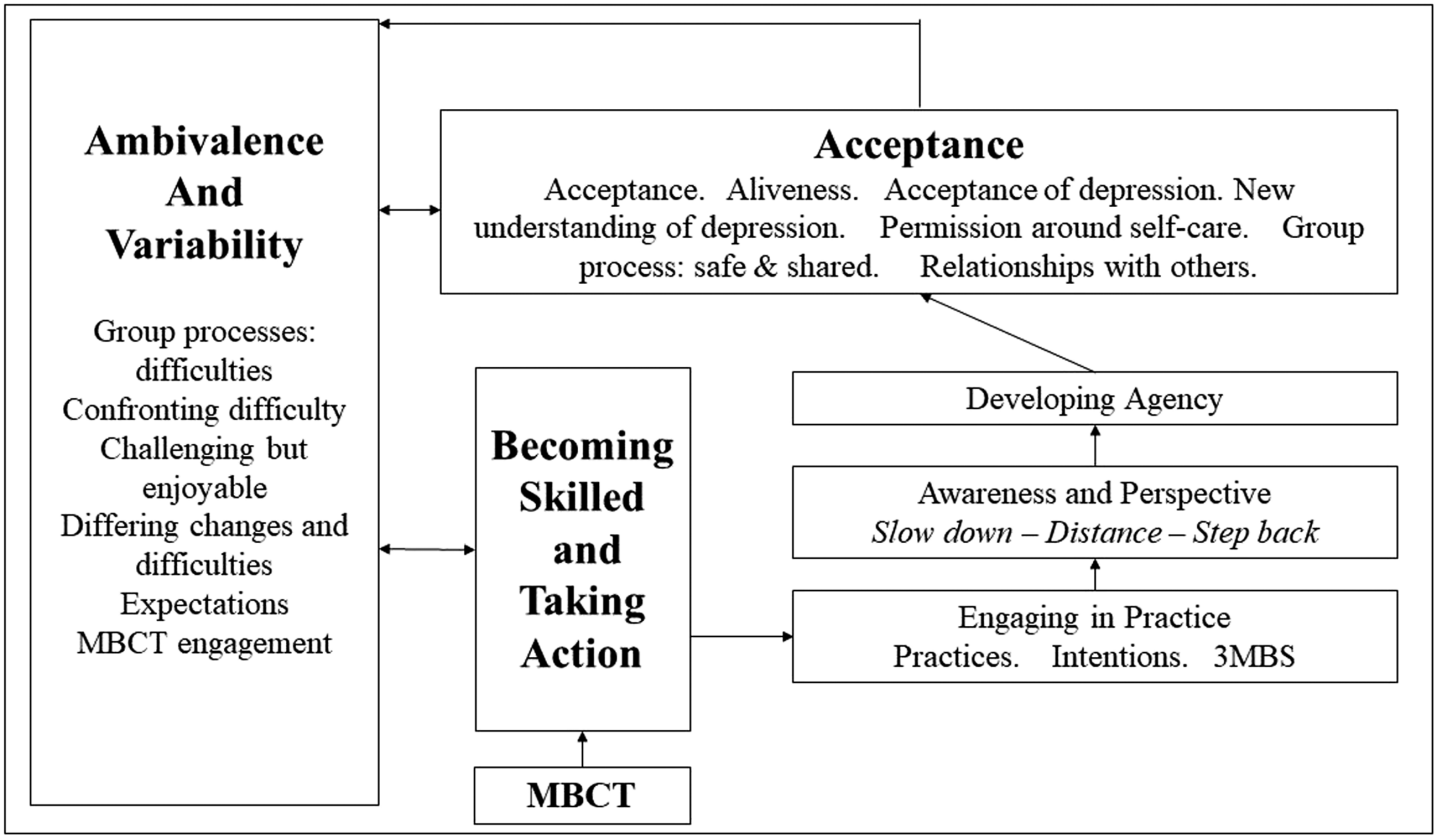
Meta‐synthesis of participants' experiences of Mindfulness‐based Cognitive Therapy (MBCT). Arrows depict either putative causal or bi‐directional pathways.

Table 3 details the synthesis results including the higher‐order, overarching themes, 18 second‐order constructs, the studies in which they appear, as well as descriptions and quotes. Figure 2 includes a visual representation of the synthesis and the proposed interactions between the overarching themes. Briefly, Figure 2 shows how participating in MBCT allowed for becoming skilled in mindfulness and taking action through engaging in mindfulness practice, developing increased awareness and perspective, as well as personal agency over experience and depression. Having developed this, participants began shifting towards acceptance of their experiences, self, others and depression. Although ambivalence and variability of experiences were present somewhat throughout the overarching themes, the core second‐order constructs focusing on ambivalence and variability were grouped together. Change within the model was not always linear with interconnections between the themes. Of note, six studies described participants' prior experiences with depression; however, we have not included these here as they do not fit with the study aims of understanding experiences of MBCT. However, the analysis is presented in Table S11.

**TABLE 3:** Overarching themes and translations

Overarching theme (third^−^order construct)	Second‐order constructs	Translation of second‐order construct	Second‐order example quote (studies*)	Studies^a^
**Becoming skilled and taking action**	Practices	Preferences for different practices.	‘Participants used both formal and informal practice on a weekly basis and had incorporated some sort of daily mindfulness exercise as an everyday habit’ (13)	1, 2, 3, 4, 5, 6, 7, 9, 10, 11, 12, 13, 16, 17, 18, 19, 21
	Intentions to practice	Either ongoing or intentions to continue practice and using skills post‐MBCT.	‘Most of the course participants continued to use some of the mindfulness exercises three months after the course ended’ (3)	2, 3, 4, 5, 6, 7, 8, 15, 18, 21
	3‐min breathing space (3MBS)	Regular engagement in the 3MBS. Proactive use of the 3MBS.	‘Participants found the 3‐min breathing space helpful’ (10)	3, 10, 16, 18
	Awareness and perspective	Increased awareness of and perspective around relationships with thoughts, emotions, sensations and depression. Slowing down, stepping back and creating distance (reduced reactivity).	Mindfulness practice ‘enabled them to calm down and step back from reacting automatically’ (11)	1, 2, 3, 4, 5, 6, 7, 9, 10, 11, 12, 13, 14, 15, 18, 19, 21
	Agency and control around depression	Shifting to a sense of agency and control around depression. Feeling skilled in understanding warning signs, relapse and recognizing the responsibility over depression this brings.	‘Gaining control and choice over thought processes felt “life‐changing” and, for some, they no longer felt they were a passive recipient or “hostage” but instead felt more active control over negative thoughts.’ (21)	1, 2, 3, 4, 5, 6, 7, 9, 11, 12, 13, 14, 15, 16, 18, 19, 21
**Acceptance**	Acceptance	Acceptance of thoughts and emotions, and what can and cannot be changed. Challenges around acceptance.	Participants ‘had learned a more accepting way to deal with experiences…in the sense of seeing themselves and their mental health as it really was’ (1)	1, 2, 3, 4, 5, 6, 7, 8, 9, 11, 13, 14, 18, 19, 21
	Aliveness	More actively involved in and enjoyment of life, feeling happier, calm and more alive.	MBCT ‘had an active focus on positive functioning, and encouraged them to take part in activities that brought happiness and joy’ (19)	1, 2, 3, 4, 5, 6, 7, 9, 11, 12, 13, 15, 18, 19, 21
	Acceptance of depression	Increased knowledge of and acceptance of depression	‘The overarching theme of acceptance incorporates a number of processes that appear to have increased their capacity to accommodate these ongoing depression‐related phenomena with less distress’ (5)	5, 13, 19
	New understanding of depression	Enhanced understanding, challenging stereotypes and shifting beliefs from a biomedical understanding of depression	Participants ‘learned a different model of depression and developed a better understanding of “how the mind works”’ (19)	1, 5, 7, 13, 18, 19
	Permission around self‐care	A move towards prioritizing the self through permission to prioritize self‐care, better communication of needs, and increased self‐compassion alongside struggles to engage in self‐care	‘Participants realised that they were not indispensable and that they could afford time to recharge’ (13)	2, 4, 5, 6, 7, 8, 9, 11, 12, 13, 14, 15, 18, 19, 21
			‘Participants reflected on a different way of relating to themselves through becoming more “compassionate”, “patient”, “kinder”, “forgiving”, and taking a “softer approach to thoughts”’ (21)	
	Group process: A safe and shared experience	Importance of others in the group in enabling safety and sharing experiences. A shared group experience helped to feel understood and supported. Importance of the MBCT instructor.	The importance of ‘being understood by the group, realising that you were not alone and being able to show emotion in a safe environment’ (3)	1, 2, 3, 4, 5, 7, 8, 10, 11, 14, 15, 16, 17, 18, 19, 20, 21
			A ‘protective environment for openly sharing with and being compassionate to each other’ (20)	
	Relationships with others	Improved closeness, bonding, communication with and emotional availability for others. Others noticing changes. Ongoing struggles or no changes.	‘They had experienced an increased ability to see that there were a number of reasons for their child's behaviour…related to increased empathy and acceptance towards themselves and their children’ (7)	2, 4, 5, 6, 7, 8, 11, 12, 13, 15, 17, 18, 20, 21
			‘Many participants emphasized the importance of community, bonding and belonging’ (20)	
**Ambivalence and variability**	Group processes: Difficulties	Group tensions, negative comparisons with others, challenges hearing others' experiences. Different opinions around the group experience.	‘Some individuals described experiencing tensions related to being in a group and a tendency to make social comparisons’ (8)	2, 3, 6, 8, 12, 15, 16, 18, 20, 21
	Confronting difficulty	Feelings of vulnerability, hopelessness and feeling out of control at times during MBCT. Struggles with some practices eliciting difficult experiences	‘Participants expressed challenges in tolerating an increase of reported symptoms of depression and anxiety’ (2)	1, 2, 3, 11, 15, 16, 21
	Challenging but enjoyable	Simultaneously difficult and enjoyable aspects of MBCT	‘There was a wish to keep going with “mindfulness” alongside recognition that this was challenging.’ (10)	1, 3, 10, 11, 16
	Differing changes and difficulties	More than more/less change, but change that was individually meaningful to participants. Depression as part of a lifelong process.	‘It was not a simple case of more or less change’ (11)	2, 5, 11, 13, 21
	Expectations	Different initial expectations and impact on outcome.	‘Those with open and flexible expectations described fewer barriers and initial negative experiences than those with rigid, and highly optimistic, ones’ (1)	1, 2, 3, 5, 6, 8, 10, 11, 12, 15, 16, 18, 19
	MBCT engagement	Many found MBCT acceptable, useful, helpful and supportive. However, there were logistical, practical and emotional impacts on attending MBCT and/or engaging in practices.	‘They found this type of intervention acceptable, and were able to comply with the demands of the course’ (18)	1, 2, 3, 4, 5, 6, 8, 10, 11, 12, 15, 16, 17, 18, 19, 21
			‘Scheduling problems (*were*) a barrier to joining the group’ (15)	

^a^ Studies: 1 = Mason and Hargreaves; 2 = Ma; 3 = Finucane and Mercer; 4 = Smith et al.; 5 = Allen et al.; 6 = Cebolla and Barrachina; 7 = Baillie et al.; 8 = Hopkins and Kuyken; 9 = Worsfold; 10 = Ames et al.; 11 = Bihari and Mullan; 12 = Boggs et al.; 13 = Lilja et al.; 14 = Murphy and Lahtinen; 15 = Di Toro; 16 = Chesin et al.; 17 = Racey et al.; 18 = Williams et al.; 19 = Tickell et al.; 20 = Canby et al.; 21 = Williams et al.

Despite some differences between the studies in terms of participant age, depression symptoms, interview timing and analysis, most second‐order constructs were present across multiple studies. Few differences were identified between participant experiences either post‐MBCT or during follow‐up; this may be because the majority of studies included analysis at either both or predominantly follow‐up timepoints. This may also suggest that participants' experiences were stable over time. However, there were some differences between study characteristics: two studies included adolescent participants, one study focused on participants' experience of reducing antidepressants and two studies respectively focused on body sensations and relationships with thoughts. These different focuses may have impacted on the synthesis and have been highlighted throughout the results.

### 3.5 Overarching theme one: Becoming skilled and taking action

#### 3.5.1 Engaging in practice

Across 19 studies, participants described their preferences for and intentions to engage with mindfulness practices including formal breathing meditations and body scans, alongside informal practices (e.g., mindful movement). A consistent finding across studies was that participants felt able to choose which style of practices they engaged with. For example, whilst some described persisting with the longer practices ‘because they allowed time for the mind to slow down and focus’ (Boggs et al., 2014), others reported engaging in shorter or more informal practices (they ‘favoured the shorter practices and tended to drop the longer ones’; Williams et al., 2018). Indeed, some participants chose not to practice formal mindfulness meditations, choosing instead to incorporate informal practices into daily ‘activities such as walking the dog or washing the dishes’ (Finucane & Mercer, 2006).

Many participants reflected on how they developed a proactive way of practicing; some ‘used mindfulness practice to help themselves to cope with crises’ (Bihari & Mullan, 2014) and used ‘mindfulness practices reactively when feeling low or stressed’ (Williams et al., 2021). However, for others it became difficult to engage in ‘when their mood was in decline’ (Lilja et al., 2015) or to believe that practices could help ‘when feeling very distressed’ and so ‘did not practice’ (Ames et al., 2014). One practice that was more easily incorporated into daily life and used proactively was the 3‐min breathing space (3MBS*), which was often referred to as a short, ‘accessible’ and ‘useful’ practice (Chesin et al., 2018; Smith et al., 2007) and noticing changes in mood as a result (‘changes in irritability and anger’; Bailie et al., 2012). This was particularly evident during follow‐up whereby engaging in practices may have become harder without the structure and support of MBCT (‘the majority continued to use the three minute breathing space’; Finucane & Mercer, 2006) and in those experiencing current symptoms. Hopkins and Kuyken (2012) who specifically focused on participants' experiences of MBCT reunion sessions during follow‐up reported that, for some, reunions enabled a reconnection with the practices and validation around the difficulty in sustaining practice beyond MBCT due to the ‘opportunity to share experiences of the challenges of maintaining regular and sustained practices’.

#### 3.5.2 Awareness and perspective

Across 17 studies, engaging in MBCT allowed for increased awareness of and a different, broader perspective on experiences. Many participants reflected on their increased awareness of depression‐specific thoughts, emotions and body sensations and particularly those ‘behaviors that signal relapse’ (Boggs et al., 2014). Developing this awareness and ‘shifts in reactivity’ (Williams et al., 2021) provided a foundation in being able to slow down, create ‘distance’ from experiences (Mason & Hargreaves, 2001), ‘stepping back’ (Ames et al., 2014) and provide an ability to ‘observe and describe their emotions and negative thought processes’ (Lilja et al., 2015). As a result, participants noticed how thoughts and feelings became less emotionally charged and more transient, as well as noticing reduced levels of both internal (e.g., to thoughts) and external reactivity (e.g., with others; Bailie et al., 2012; Bihari & Mullan, 2014; Lilja et al., 2015).

Many studies highlighted participants' different relationships with rumination, now feeling able to notice the subtleties of rumination, feeling less ‘attached to thoughts’ (Di Toro, 2016), spending ‘less time ruminating’ (Bailie et al., 2012; Cebolla i Martí & Miró Barrachina, 2009; Smith et al., 2007), gaining ‘control over … tendencies to catastrophise and ruminate’ (Williams et al., 2021) and feeling ‘able to break these destructive thought cycles’ (Murphy & Lahtinen, 2015). In one study which focused specifically on body awareness, participants commented on a sense of increased control over their body, recognizing its power over reducing attachment to negative thinking (mindful walking engaged ‘thinking, leaving less room for unpleasant thoughts’; Worsfold, 2013). The relationship with rumination seemed less about ‘an absence of threatening thoughts’ but signalled a change in awareness of and engagement with thoughts, with participants feeling more skilled in being able to ‘manage and negotiate around and away from’ (Murphy & Lahtinen, 2015) rumination.

#### 3.5.3 Developing agency

Many participants across 17 studies subsequently spoke about noticing shifts from a passive to a more active ‘sense of personal agency’ and control over depression, warning signs, (e.g., Allen et al., 2009; Boggs et al., 2014; Chesin et al., 2018) and antidepressant use in Tickell et al. (2020). As a result of increased agency, some felt they could ‘identify with depression with less distress’ (Allen et al., 2009) and held a better understanding of warning signs, which may signify an impending relapse (‘developing awareness of personal indicators of worsening mental state’; Mason & Hargreaves, 2001). However, for some, this shift came with a sense of responsibility and ‘unhelpful pressure’, preferring instead to have a ‘guarantee’ of antidepressants as the active agent in their depression (Tickell et al., 2020).

### 3.6 Overarching theme two: Acceptance

#### 3.6.1 Accepting experiences and depression

Through engagement in MBCT, and having developed changes in awareness, perspective and agency, participants across 15 studies described acceptance towards their depression‐related thoughts and emotions, noticing the ability to ‘accept thoughts and feelings’ (Allen et al., 2009), ‘allowing’ space for difficult or unpleasant emotions (Worsfold, 2013) without feeling overwhelmed, and feeling able to ‘turn towards’ body sensations and pain (Lilja et al., 2015). Participants developed acceptance of the present moment, moving away from pulls into negative thoughts or worries and ‘letting go of anxiety and re‐discovering joy’ (Finucane & Mercer, 2006). Some participants reflected on an increased ‘ability to reconnect with their “old self”’ (Williams et al., 2021) and that acceptance of their experiences had helped participants to feel ‘vividly alive’ and ‘energetic’ (both Smith et al., 2007).

Three studies specifically described shifts towards acceptance of a lifelong difficulty with depression, through shifts away from a ‘negative’ depressed identity to acceptance of depression as ‘an aspect of human experience’ (Tickell et al., 2020). However, acceptance of depression was sometimes challenging as acceptance implied a shift from an illness model of depression towards having responsibility and control over depression (‘its presence no longer expresses an external contingency (for which a person cannot be blamed) but an internal contingency’; Allen et al., 2009). Similarly, others reported difficulty with accepting a psychological versus a biomedical understanding of depression, leading to more self‐blame during experiences of low mood or relapse (‘when people developed a psychological understanding and then went on to relapse, they blamed themselves’; Tickell et al., 2020). As a result, some participants expressed desires to stay with a biomedical understanding (Tickell et al., 2020).

#### 3.6.2 Permission for self‐care

Fifteen studies described shifts in self‐acceptance. Studies described how participants developed acceptance and responsibility to take care of themselves and ‘prioritise their own needs before others’ (Lilja et al., 2015). Some could reframe self‐care as a ‘necessary part of their ongoing recovery’, no longer seeing self‐care as ‘fluffy’ (both quotes from Tickell et al., 2020). With permission to engage in self‐care, participants noticed increased self‐compassion, ‘self‐esteem’ (Cebolla i Martí & Miró Barrachina, 2009) and feeling better able to ‘accept their own fallibilities … without judgement’ (Murphy & Lahtinen, 2015). Of course, acceptance was not easy and these changes varied amongst participants experiencing ongoing ‘guilt or self‐blame’ (Allen et al., 2009) and how the ‘intensity and power of such *(self‐critical)* thoughts and emotions was lessening’ (Williams et al., 2021), with some describing their experience as being ‘almost with compassion’ (Murphy & Lahtinen, 2015) in recognition of a gradual, ongoing change towards self‐compassion.

#### 3.6.3 A shared group experience

Across 17 studies, participants described how the MBCT group and its facilitators helped to foster a ‘safe’ and ‘shared’ experience (Williams et al., 2021) in which participants felt able to bond, a ‘sense of community’, and develop a sense of closeness, trust and belonging, in which to express vulnerabilities (Canby et al., 2021). Specifically, participants described feeling ‘understood’, cared for and feeling ‘able to show emotion in a safe environment’ (Finucane & Mercer, 2006). A number of participants commented on how being able to share and identify ‘with other people who suffer depression’ (Allen et al., 2009) contributed to a reframing of a depressed identity and increased acceptance of depression through reduction of isolation and stigma (‘no longer feeling alone or different’; Bihari & Mullan, 2014). One study reported on the importance of the MBCT instructor, and their relationship with participants (‘instructors made them feel comfortable to self‐disclose or participants felt cared for’; Canby et al., 2021).

#### 3.6.4 Relationships with others

Across 14 studies, and particularly in Bihari and Mullan (2014), many participants reported changes in interpersonal relationships following MBCT. For example, participants reported more relaxed and loving relationships, increased intimacy (‘greater emotional closeness’; Allen et al., 2009), empathy, ‘more mindful, responsive communication’ (Bihari & Mullan, 2014) and ‘feeling better able to relate to others in a “healthier” way’ (Williams et al., 2021). Many participants described how owing to a better ability to relate differently to their experiences and increased self‐acceptance, they felt less judgemental of others' feelings and perspectives (‘relating mindfully to their own experiences … led to increased understanding … in their relationships’; Bihari & Mullan, 2014). Participants noted feeling more accepted by others, feeling ‘better able to talk about their depression with their family and friends’ and how others had ‘noticed changes’ in them (Ma, 2002; Smith et al., 2007). In one study focused on the parenting relationship, participants described ‘increased bonding’ with and having more ‘emotional availability’ for their children (Bailie et al., 2012). Similarly, from the child's perspective, Racey et al. (2018) described how young people felt more able to approach and engage with their parents (‘improved relationships within the wider family’).

In contrast, some participants reported that MBCT ‘had no effect on their relationships’ (Allen et al., 2009) with others reporting ‘continuing difficulties’ and ‘feelings of frustration’ in their relationships, although for some this may have been due to less engagement with mindfulness practice (all Bailie et al., 2012). It was noticeable how the only study delivering online MBCT with no live group option did not report changes in interpersonal relationships (‘little evidence of themes indicating improved relationships’; Boggs et al., 2014), suggesting an important relationship between engaging in group‐based MBCT and interpersonal changes.

### 3.7 Overarching theme three: Ambivalence and variability

#### 3.7.1 Group processes: Difficulties

Although being in a group gave some participants a sense of safety and security, 11 studies described how being in a group prompted difficult feelings. Specifically, being in a group meant that some participants found it difficult to relate to and connect with others (Canby et al., 2021), or made negative ‘social comparisons’ and found it ‘challenging’ to be with others who are or have been depressed (Hopkins & Kuyken, 2012). Additionally, listening to others' experiences sometimes felt triggering if ‘discussion turned to suicidal behavior or interpersonal loss’ (Chesin et al., 2018). In contrast, in the study delivering online MBCT, some preferred the flexibility and reduced pressure of an online, no‐group format, indicating that they were not ‘group learners’ (Boggs et al., 2014).

#### 3.7.2 Differing changes and difficulties

Across seven studies, participants reflected on conflicting and difficult feelings resulting from mindfulness practices whereby some allowed for a ‘safe space to explore difficult emotions’ (Williams et al., 2021) and some mindfulness practices led to ‘increase(*d*) feelings of vulnerability’ or ‘hopelessness’ (all quotes Bihari & Mullan, 2014). While for some participants these ‘experiences were described as transient’ (Chesin et al., 2018), they were not transient for everyone and engaging in mindfulness‐based practices subsequently led to more distressing experiences. This was especially highlighted by Finucane and Mercer (2006) whereby two participants with previous traumatic experiences found that engaging in the body scan prompted intense, painful memories and/or flashbacks. One participant felt able to adapt their mindfulness practice and chose to engage in a 3MBS instead having found it ‘a useful exercise and continued to use it regularly’. Another participant, with experiences of childhood sexual abuse, enjoyed engaging with the body scan and ‘continued to practice the longer meditation practices several times a week’ (Finucane & Mercer, 2006). In five studies, participants described a contrast between how MBCT could prompt difficult thoughts or feelings but commented that MBCT was still ‘enjoyable and beneficial albeit challenging’ (Bihari & Mullan, 2014) because of the benefits arising from it. Finally, five studies specifically described how change varied between participants and that it was less about a quantifiable or ‘one single path to change’ (Ma, 2002). Instead, change was viewed as individually meaningful to participants' lives (‘it was not simply a case of participants experiencing more or less change’; Bihari & Mullan, 2014) and as ‘a gradual change to what are often lifelong patterns of relating to experience, self, and others’ (Williams et al., 2021).

#### 3.7.3 Expectations and engagement

Thirteen studies described participants' initial expectations for MBCT and, for some, the subsequent impact on their engagement with MBCT. Some participants had hoped for a ‘cure’ to take away their depression (Mason & Hargreaves, 2001; Tickell et al., 2020) or held pre‐existing ‘high expectations’ (Finucane & Mercer, 2006). For some, their experience of MBCT did not meet their expectations and they had hoped ‘to receive more CBT’ (Hopkins & Kuyken, 2012) or wanted longer sessions (‘most of the group would have liked … another 3–4 weeks’; Finucane & Mercer, 2006). Others had expressed apprehension around participating in a group or were ‘unsure’ or sceptical about mindfulness (Chesin et al., 2018). Those who held open‐minded expectations subsequently reported ‘fewer barriers’ to engagement (Mason & Hargreaves, 2001). However, for those holding either ‘unrealistically positive’ or ‘very negative’ expectations, these became a barrier for engagement in MBCT (Tickell et al., 2020). Even where MBCT fell short of their expectations, some participants persisted with MBCT because of their original motivations in reducing or avoiding medication or their motivation to address ‘the chronicity of their problems with anxiety and depression’ (Finucane & Mercer, 2006).

Across 16 studies, MBCT was described as ‘acceptable’ (e.g., Ames et al., 2014; Chesin et al., 2018; Racey et al., 2018), helpful and an ‘enjoyable, beneficial experience’ (Bihari & Mullan, 2014), particularly in terms of the support provided by the facilitators and the group (Smith et al., 2007; Williams et al., 2018). However, some participants described a ‘consistent conflict between the desire or inclination to avoid versus engage, particularly in mindfulness practices and MBCT itself’ (Williams et al., 2021). Some reported a range of difficulties impacting on their ability to engage in MBCT and the mindfulness practices. Difficulties included practical and logistical impacts on getting to the MBCT (e.g., childcare commitments and transport) and physical limitations (e.g., pain). Finding both the physical and mental space to accommodate the mindfulness practices became difficult once MBCT had finished, whereby it became ‘difficult for people to engage in an approach that required time and effort’ (Tickell et al., 2020).

## 4 DISCUSSION

Across 21 qualitative studies, we aimed to systematically review and synthesize the experiences of MBCT for people with depression. Using reciprocal translation, we reported 18 second‐order constructs that appeared within and across the 21 studies. Using a line of argument synthesis, we developed three overarching themes (third‐order constructs): ‘Becoming skilled and taking action’, ‘Acceptance’ and ‘Ambivalence and variability’.

Our findings replicate and extend the findings of the earlier meta‐syntheses (Cairns & Murray, 2013; Malpass et al., 2012; Wyatt et al., 2014) in terms of understanding MBCT and participants' accounts of increasing awareness, control, relating differently to experience, relationships and struggles. However, our findings contribute further towards understanding MBCT more specifically for people with depression and highlight the importance of developing a sense of agency and the processes involved in developing this (e.g., engaging in practice, awareness and agency). Further, our meta‐synthesis positions ‘acceptance’ as an overarching theme encompassing many changes in the relationship with depression, self and others, while enabling feelings of vitality and ‘aliveness’. Finally, ‘ambivalence and variability’ was positioned as a key theme which, although reported in other meta‐syntheses, was not previously identified as a core theme. MBCT is recommended by NICE guidelines for both recurrent and less severe current episodes of depression (National Institute for Health and Care Excellence, 2022) and sequential models for the treatment of depression have highlighted the important roles that both pharmacotherapy and psychotherapy play in reducing risk to relapse in depression (Cosci et al., 2020; Guidi et al., 2016). Each of the themes in our paper contributes further to the broader understanding of how MBCT works to reduce the risk of relapse in depression.

The first overarching theme ‘becoming skilled and taking action’ highlighted three cumulative and interrelated levels beginning with engaging in mindfulness practice, to developing awareness and perspective, and subsequent agency and control over experience. Quantitative studies have also reported increases in attention and awareness following MBCT (Van Den Hurk et al., 2012) as well as changes in reactivity and rumination (Britton et al., 2012; van der Velden et al., 2015; van Vugt et al., 2012); however, studies have generally reported less around agency and control. This overarching theme reflects the core aims of MBCT as through engagement in and development of an experiential understanding of mindfulness practices and principles, participants develop a greater awareness of all experiences (e.g., thoughts, emotions and sensations; Segal et al., 2013), including automatic or ingrained processes such as rumination, self‐criticism and experiential avoidance (Alloy et al., 2018; Cantazaro & Wei, 2010; Cribb et al., 2006; Hayes, 2004; Koster et al., 2011; Mongrain & Leather, 2006; Segal et al., 2013; Watkins, 2008). Further, developing a sense of personal agency and control can be a particularly meaningful change in those experiencing depression where they may have previously felt powerless or hopeless (Gilbert, 2017).

The second overarching theme described acceptance towards a range of experience, depression, self and others, enabling an increased sense of aliveness, self‐care and improved relationships. Acceptance and principles of mindfulness are closely linked, whereby engaging in mindfulness and adopting a non‐judgemental and curious attitude to experience can lead to a re‐framing of experience (e.g., thoughts) as transient mental events rather than as truths (Cavanagh et al., 2014). Developing acceptance may also enable an ability to step back from getting caught up in difficulties (Crane, 2009). Our findings are in line with the aims and quantitative studies of MBCT whereby MBCT encourages a shift towards letting go of judgements and reactive, automatic ways of responding, whilst developing a kinder, more compassionate approach to experience, including self and others (Crane, 2009; Segal et al., 2013; van der Velden et al., 2015). Our findings also suggest that through belonging to a safe group in which to share feelings and experiences with depression, this may have encouraged self‐acceptance and acceptance from others, particularly as disclosing personal inadequacies can lead to increased acceptance by others in a therapy group (Yalom, 1995).

Our third overarching theme of ‘ambivalence and variability’ incorporated different experiences around taking part in a group and being with others, feelings towards the self and difficult experiences. This theme also incorporated participants' expectations around taking part, as well as contrasts between participants finding MBCT to be an acceptable intervention and those who reported practical or logistical impacts on engagement. The poor fit between initial expectations and reality may contribute to ambivalence about MBCT as a useful approach. Expectations play an important role in future outcome, where client expectations for therapy have been consistently shown to impact therapy outcome (Constantino et al., 2018; Cuijpers et al., 2019; Wampold, 2015). The relationship between expectations and outcome may be more complex for participants with long histories of depression, however, given that a higher number of ‘episodes’ of depression has shown to be negatively correlated with initial expectations for change (Vîslă et al., 2019).

For some, engaging in MBCT was sometimes challenging as MBCT invites participants to turn towards difficult or painful experiences that might usually be avoided (e.g., difficult thoughts, emotions and sensations; Crane et al., 2014; Segal et al., 2013). Indeed, many participants across the studies were able to deliberately make a choice as to which mindfulness‐based practices they engaged in both during and beyond MBCT, be it the more traditional ‘formal’ practices (e.g., mindfulness of breathing) or the more ‘informal’ practices (e.g., cooking, cleaning and movement). The informal practices may have felt more accessible to participants, particularly in times of distress or low mood, and indeed, some participants were able to adapt their choice of practices as a result.

However, for some participants, engaging in mindfulness‐based practices contributed to an increase in feelings of vulnerability, hopelessness and re‐triggering of painful memories and/or flashbacks. For others, being in a group with other people who have experiences of depression was sufficient to trigger difficult feelings and memories of their own experiences of depression. Although there is an emerging literature around the potential for adverse effects of mindfulness meditation (Lindahl et al., 2017) and of mindfulness‐based practices found in interventions like MBCT (Baer et al., 2021), there is still much that we do not know. Thus, it is important to bear in mind that experiences during and beyond MBCT will differ between participants and that whilst some unpleasant or negative experiences may be expected during MBCT, there is also the potential for adverse effects which may be more long‐lasting (e.g., increased anxiety and depression, dissociation, derealisation and/or depersonalisation, emotional numbing, hyperarousal (e.g., panic and agitation) and re‐experiencing of traumatic memories; Baer et al., 2021; Britton et al., 2021).

### 4.1 Clinical implications

Our study highlights a number of clinical implications. Firstly, MBCT teachers should incorporate the overall findings of this review when discussing the possible benefits of engaging in mindfulness practice (e.g., increasing awareness, perspective, agency and acceptance) with participants. All MBCT teachers, whether in clinical, community or research settings, should ensure that participants are given full opportunity to discuss their expectations of MBCT and whether these can be realistically met during MBCT, to ensure fully informed participation. Further, when facilitating MBCT for people with histories of depression, it is arguably more important to consider the role of expectations. MBCT teachers should maintain an awareness that their clients may hold potentially higher levels of hopelessness and lower expectations of change at the start, during and at the end of MBCT. We encourage MBCT teachers to find a balance between acknowledging the realistic expectations of what MBCT can and cannot offer, whilst also aiming to instil hope in clients as they navigate through the MBCT course. MBCT teachers should discuss the possible logistical, practical and emotional challenges that may arise with taking part and offer further support or alternative options for attendance (e.g., remote methods).

Given our findings of the importance of the development of agency and control over thoughts, feelings and experiences, and permission for self‐care, MBCT teachers should work to empower clients who may typically have a strong history of feeling hopeless or powerless. Specifically, during MBCT sessions, MBCT teachers can help their clients to develop a sense of metacognitive awareness of their thoughts, feelings and experiences through explicitly naming and increasing awareness of how they are beginning to recognize and develop their own agency and control. MBCT teachers could actively monitor group dynamics and offer more regular check‐ins regarding the group sense of cohesion and safety, particularly as our findings showed that the belonging to a safe group enabled self‐acceptance and acceptance from others. This may be particularly important for those who are currently experiencing symptoms of depression as our findings showed that this was a more commonly mentioned theme across those studies investigating participants with current symptoms.

Additionally, MBCT teachers and participants should discuss their expectations for what may feel triggering (e.g., participants hearing discussions around suicide) or what might arise during a practice (e.g., painful memories and flashbacks). It will be important that individuals wishing to undertake MBCT are fully assessed in terms of their clinical histories, with an emphasis on explicitly discussing the potential for memories, flashbacks, feelings and thoughts to be triggered during *both* mindfulness practices and class discussions. If individuals subsequently decide to still continue with MBCT, it will be important that teachers and participants discuss ways in which support could be offered to participants during MBCT practices, class discussions and in their homework practice. This may involve inclusion of content warnings prior to delivered content or group discussions or explicitly giving permission to participants to pause or sit out of practices or discussions. This may also involve an in‐class discussion around the potential to feel triggered by difficult practices or conversations and providing additional guidance at those times: ad hoc practices around sitting with difficulty or inviting pauses for acknowledgement of the difficulty and group reflection.

As mentioned in the results, most second‐order constructs were present across multiple studies, which supports the strength of the overarching themes as participants reported changes in line with the underlying principles of MBCT, suggesting that MBCT is working as intended. However, it could also be argued that there may be some questions that are not explored or some participant experiences that are missed; this may be the case for those participants who drop out or do not sign up to an MBCT group. Exploring these perspectives in future research would help to clarify this. Further research could specifically explore how experiences are shaped by current depression severity alongside the core themes. Our findings highlight the importance of considering the qualitative experiences of MBCT, especially in light of our findings around variable change that is individually meaningful, as meaning becomes a difficult concept to quantify.

### 4.2 Limitations of the included studies

Thirteen studies did not specifically acknowledge the impact of the participant–researcher relationship; thus, we cannot be certain what effect this may have had on the original authors' interpretations. A number of studies included researchers who have been extensively involved in MBCT practice and research whose particular biases, expectations and conflicts of interest may have impacted on study findings. However, the inclusion of studies, which were conducted in non‐expert MBCT centres of research works to mitigate this. There were a number of similarities across the different study characteristics. However, it is noticeable that the concept of ‘acceptance’ was rarely, if at all, discussed within studies from the United States, in comparison to those from the United Kingdom/Europe. These findings may be a result of cultural implications, which may be worth exploring in further research. There were significantly fewer studies focused on adolescent and older adult experiences, where there may also be different considerations and experiences. Gender distribution within studies was skewed towards participants identifying as female. This might be explained by higher proportions of women experiencing depression (Kuehner, 2017) or suggest that MBCT is initially attractive to people identifying as female, but it warrants further investigation into the experiences of MBCT for people identifying as male. Finally, it should be noted that all studies were conducted across the United Kingdom, mainland Europe and the United States. Therefore, there may be important considerations around the impact of additional demographic and social factors (such as race or income) on the presence of and severity of depressive symptoms as well as the treatment effects of MBCT. This may further highlight an important area for future research to explore the demographics of who feels able to take part in MBCT, particularly in regards to racial trauma and inequality (Ahsan, 2020).

### 4.3 Limitations of the meta‐synthesis

The lead researcher was interested in and experienced in delivering and researching MBCT, which may have impacted on the interpretations made in this synthesis. However, efforts to stay aware of and reflect on this were made through use of the reflective log and in‐team discussions. All studies were required to be written in English which may have meant that we missed some studies. The lead researcher screened all of the titles and abstracts, of which an independent researcher screened 15%, with good agreement between the two researchers' search strategies. Additionally, the lead researcher conducted the coding of the individual studies, which was discussed regularly with the other co‐authors to ensure resolution of any disagreements. It should be acknowledged that in order to enhance researcher objectivity and reduce the chance for researcher error, it would have been beneficial to have had a second researcher conduct both a parallel screening of all the titles and abstracts and the coding of the individual studies. Finally, although unpublished theses and conference abstracts were detected by our search strategy, we did not specifically search any grey literature databases thus may have missed other relevant studies.

## 5 CONCLUSION

Across 21 qualitative studies, we found three overarching themes specifically relevant to participants' experiences of MBCT, including ‘Becoming skilled and taking action’, ‘Acceptance’ and ‘Ambivalence and Variability’. Our findings suggest that whilst some people benefit from developing skills in mindfulness, develop awareness, agency and acceptance of themselves and others, others may have difficulty in practicing mindfulness and attending MBCT classes. This may indicate that MBCT needs to be carefully tailored to individual experiences and expectations in order to maximize relapse prevention. Our findings provide an update and a more specifically tailored meta‐synthesis of qualitative studies for participants with depression participating in MBCT. Our findings help to enhance confidence in MBCT, to further understand the processes involved in change following MBCT and to better understand who may experience difficulties.

## CONFLICT OF INTEREST

The study authors have no conflicts of interest to declare.

## Supporting information


**Table S1.** PRISMA checklist
**Table S2.** PRISMA 2020 for Abstracts checklist (Page et al., 2021)
**Table S3.** eMERGE guidance
**Table S4.** Dates for checking additional sources
**Table S5.** Meta‐ethnography steps
**Table S6.** Step 4. First‐order categories across studies incorporating author themes, subthemes, descriptions and interpretations (chronological order)
**Table S7.** Sensitivity analyses based on study characteristics and organized by overarching themes and second‐order constructs
**Table S8.** Step 4. 2nd order categories across studies incorporating author themes, subthemes, descriptions and interpretations
**Table S9.** Full text screening decisions
**Table S10.** CASP quality results by study
**Table S11.** Second‐order construct and example quote

## Data Availability

Data sharing is not applicable to this article as no new data were created or analysed in this study.
