# Supplementary material for: A systematic review and meta‐ethnographic synthesis of Mindfulness‐based Cognitive Therapy for people with major depression
Source: Clin Psychol Psychother. 2022 Aug 15;29(5):1494–514. doi: 10.1002/cpp.2773 (PMC9805101; doi:10.1002/cpp.2773)
Supplement: Supplementary file 1 — Table S1. PRISMA checklist Table S2. PRISMA 2020 for Abstracts checklist (Page et al., 2021) Table S3. eMERGE guidance Table S4. Dates for checking additional sources Table S5. Meta‐ethnography steps Table S6. Step 4. First‐order categories across studies incorporating author themes, subthemes, descriptions and interpretations (chronological order) Table S7. Sensitivity analyses based on study characteristics and organized by overarching themes and second‐order constructs Table S8. Step 4. 2nd order categories across studies incorporating author themes, subthemes, descriptions and interpretations Table S9. Full text screening decisions Table S10. CASP quality results by study Table S11. Second‐order construct and example quote [file CPP-29-1494-s001.docx]

**Supplementary Material**

**Supplementary Material One**

**PRISMA Checklists**

These checklists are taken from the recent publication by Page et al. (2021) who have updated the PRISMA 2009 statement and advise that these two checklists below should now be used instead.

**Table 1.** PRISMA checklist

| **Section and Topic** | **Item #** | **Checklist item** | **Location** |
| --- | --- | --- | --- |
| **TITLE** | | |  |
| Title | 1 | Identify the report as a systematic review. | Title |
| **ABSTRACT** | | |  |
| Abstract | 2 | See the PRISMA 2020 for Abstracts checklist. | Supplementary Material |
| **INTRODUCTION** | | |  |
| Rationale | 3 | Describe the rationale for the review in the context of existing knowledge. | Introduction |
| Objectives | 4 | Provide an explicit statement of the objective(s) or question(s) the review addresses. | Introduction |
| **METHODS** | | |  |
| Eligibility criteria | 5 | Specify the inclusion and exclusion criteria for the review and how studies were grouped for the syntheses. | Method |
| Information sources | 6 | Specify all databases, registers, websites, organisations, reference lists and other sources searched or consulted to identify studies. Specify the date when each source was last searched or consulted. | Method Supplementary Material |
| Search strategy | 7 | Present the full search strategies for all databases, registers and websites, including any filters and limits used. | Method |
| Selection process | 8 | Specify the methods used to decide whether a study met the inclusion criteria of the review, including how many reviewers screened each record and each report retrieved, whether they worked independently, and if applicable, details of automation tools used in the process. | Method |
| Data collection process | 9 | Specify the methods used to collect data from reports, including how many reviewers collected data from each report, whether they worked independently, any processes for obtaining or confirming data from study investigators, and if applicable, details of automation tools used in the process. | Method |
| Data items | 10a | List and define all outcomes for which data were sought. Specify whether all results that were compatible with each outcome domain in each study were sought (e.g. for all measures, time points, analyses), and if not, the methods used to decide which results to collect. | Method |
|  | 10b | List and define all other variables for which data were sought (e.g. participant and intervention characteristics, funding sources). Describe any assumptions made about any missing or unclear information. | Method |
| Study risk of bias assessment | 11 | Specify the methods used to assess risk of bias in the included studies, including details of the tool(s) used, how many reviewers assessed each study and whether they worked independently, and if applicable, details of automation tools used in the process. | Method |
| Effect measures | 12 | Specify for each outcome the effect measure(s) (e.g. risk ratio, mean difference) used in the synthesis or presentation of results. | N/A |
| Synthesis methods | 13a | Describe the processes used to decide which studies were eligible for each synthesis (e.g. tabulating the study intervention characteristics and comparing against the planned groups for each synthesis (item #5)). | Supplementary Material |
|  | 13b | Describe any methods required to prepare the data for presentation or synthesis, such as handling of missing summary statistics, or data conversions. | Method |
|  | 13c | Describe any methods used to tabulate or visually display results of individual studies and syntheses. | Method |
|  | 13d | Describe any methods used to synthesize results and provide a rationale for the choice(s). If meta-analysis was performed, describe the model(s), method(s) to identify the presence and extent of statistical heterogeneity, and software package(s) used. | Method |
|  | 13e | Describe any methods used to explore possible causes of heterogeneity among study results (e.g. subgroup analysis, meta-regression). | Method |
|  | 13f | Describe any sensitivity analyses conducted to assess robustness of the synthesized results. | Method Supplementary Material |
| Reporting bias assessment | 14 | Describe any methods used to assess risk of bias due to missing results in a synthesis (arising from reporting biases). | Method |
| Certainty assessment | 15 | Describe any methods used to assess certainty (or confidence) in the body of evidence for an outcome. | Method |
| **RESULTS** | | |  |
| Study selection | 16a | Describe the results of the search and selection process, from the number of records identified in the search to the number of studies included in the review, ideally using a flow diagram. | Results |
|  | 16b | Cite studies that might appear to meet the inclusion criteria, but which were excluded, and explain why they were excluded. | Results  Supplementary Material |
| Study characteristics | 17 | Cite each included study and present its characteristics. | Results |
| Risk of bias in studies | 18 | Present assessments of risk of bias for each included study. | Results |
| Results of individual studies | 19 | For all outcomes, present, for each study: (a) summary statistics for each group (where appropriate) and (b) an effect estimate and its precision (e.g. confidence/credible interval), ideally using structured tables or plots. | Results |
| Results of syntheses | 20a | For each synthesis, briefly summarise the characteristics and risk of bias among contributing studies. | Results |
|  | 20b | Present results of all statistical syntheses conducted. If meta-analysis was done, present for each the summary estimate and its precision (e.g. confidence/credible interval) and measures of statistical heterogeneity. If comparing groups, describe the direction of the effect. | NA |
|  | 20c | Present results of all investigations of possible causes of heterogeneity among study results. | Contained within Results |
|  | 20d | Present results of all sensitivity analyses conducted to assess the robustness of the synthesized results. | Results  Supplementary Material |
| Reporting biases | 21 | Present assessments of risk of bias due to missing results (arising from reporting biases) for each synthesis assessed. | Results |
| Certainty of evidence | 22 | Present assessments of certainty (or confidence) in the body of evidence for each outcome assessed. | Results |
| **DISCUSSION** | | |  |
| Discussion | 23a | Provide a general interpretation of the results in the context of other evidence. | Discussion |
|  | 23b | Discuss any limitations of the evidence included in the review. | Discussion |
|  | 23c | Discuss any limitations of the review processes used. | Discussion |
|  | 23d | Discuss implications of the results for practice, policy, and future research. | Discussion |
| **OTHER INFORMATION** | | |  |
| Registration and protocol | 24a | Provide registration information for the review, including register name and registration number, or state that the review was not registered. | Title page  Method |
|  | 24b | Indicate where the review protocol can be accessed, or state that a protocol was not prepared. | Title page  Method |
|  | 24c | Describe and explain any amendments to information provided at registration or in the protocol. | NA |
| Support | 25 | Describe sources of financial or non-financial support for the review, and the role of the funders or sponsors in the review. | End of manuscript |
| Competing interests | 26 | Declare any competing interests of review authors. | End of manuscript |
| Availability of data, code and other materials | 27 | Report which of the following are publicly available and where they can be found: template data collection forms; data extracted from included studies; data used for all analyses; analytic code; any other materials used in the review. | Supplementary Material |

**Table 2.** PRISMA 2020 for Abstracts checklist (Page et al., 2021)

| **Section and Topic** | **Item #** | **Checklist item** | **Present?** |
| --- | --- | --- | --- |
| Title | | |  |
| Title | 1 | Identify the report as a systematic review. | Yes (main title and in the method) |
| Background | | |  |
| Objectives | 2 | Provide an explicit statement of the main objective(s) or question(s) the review addresses. | Yes |
| Methods | | |  |
| Eligibility criteria | 3 | Specify the inclusion and exclusion criteria for the review | No, due to space limitations. However we specify that 19 studies met the study criteria. |
| Information sources | 4 | Specify the information sources (e.g. databases, registers) used to identify studies and the date when each was last searched. | Yes |
| Risk of bias | 5 | Specify the methods used to assess risk of bias in the included studies | Yes |
| Synthesis of results | 6 | Specify the methods used to present and synthesise results. | Yes |
| Results | | |  |
| Included studies | 7 | Give the total number of included studies and participants and summarise relevant characteristics of studies. | Yes |
| Synthesis of results | 8 | Present results for main outcomes, preferably indicating the number of included studies and participants for each. If meta-analysis was done, report the summary estimate and confidence/credible interval. If comparing groups, indicate the direction of the effect (i.e. which group is favoured). | Yes |
| Discussion | | |  |
| Limitations of evidence | 9 | Provide a brief summary of the limitations of the evidence included in the review (e.g. study risk of bias, inconsistency and imprecision). | Yes |
| Interpretation | 10 | Provide a general interpretation of the results and important implications. | Yes |
| Other | | |  |
| Funding | 11 | Specify the primary source of funding for the review. | No, but this has been included at the end of the manuscript as per the target journal requirements |
| Registration | 12 | Provide the register name and registration number. | Yes |

**Supplementary Material Two**

**Table 3.** eMERGE guidance

|  | | | **Reported:** |
| --- | --- | --- | --- |
| **Phase 1—Selecting meta‐ethnography and getting started** | | |  |
| Introduction | | | |
| 1 | Rationale and context for the meta‐ethnography | Describe the gap in research or knowledge to be filled by the meta‐ethnography, and the wider context of the meta‐ethnography | Introduction |
| 2 | Aim(s) of the meta‐ethnography | Describe the meta‐ethnography aim(s) | Introduction |
| 3 | Focus of the meta‐ethnography | Describe the meta‐ethnography review question(s) (or objectives) | Introduction |
| 4 | Rationale for using meta‐ethnography | Explain why meta‐ethnography was considered the most appropriate qualitative synthesis methodology | Introduction |
| **Phase 2—Deciding what is relevant** | | |  |
| Methods | | | |
| 5 | Search strategy | Describe the rationale for the literature search strategy | Method |
| 6 | Search processes | Describe how the literature searching was carried out and by whom | Method |
| 7 | Selecting primary studies | Describe the process of study screening and selection, and who was involved | Method |
| Findings | | | |
| 8 | Outcome of study selection | Describe the results of study searches and screening | Results |
| **Phase 3—Reading included studies** | | |  |
| Methods | | | |
| 9 | Reading and data extraction approach | Describe the reading and data extraction method and processes | Method |
| Findings | | |  |
| 10 | Presenting characteristics of included studies | Describe characteristics of the included studies | Results |
| **Phase 4—Determining how studies are related** | | |  |
| Methods | | |  |
| 11 | Process for determining how studies are related | Describe the methods and processes for determining how the included studies are related: | Method |
|  |  | ‐ Which aspects of studies were compared | Method |
|  |  | **AND** |  |
|  |  | ‐ How the studies were compared | Method |
| Findings | | | |
| 12 | Outcome of relating studies | Describe how studies relate to each other | Results |
| **Phase 5—Translating studies into one another** | | |  |
| Methods | | | |
| 13 | Process of translating studies | Describe the methods of translation**:** | Method |
|  |  | ‐ Describe steps taken to preserve the context and meaning of the relationships between concepts within and across studies | Method |
|  |  | ‐ Describe how the reciprocal and refutational translations were conducted | Method |
|  |  | ‐ Describe how potential alternative interpretations or explanations were considered in the translations | Method |
| Findings | | |  |
| 14 | Outcome of translation | Describe the interpretive findings of the translation. | Results |
| **Phase 6—Synthesizing translations** | | |  |
| Methods | | | |
| 15 | Synthesis process | Describe the methods used to develop overarching concepts (“synthesized translations”) | Method |
|  |  | Describe how potential alternative interpretations or explanations were considered in the synthesis | Method |
| Findings | | | |
| 16 | Outcome of synthesis process | Describe the new theory, conceptual framework, model, configuration, or interpretation of data developed from the synthesis | Results |
| **Phase 7—Expressing the synthesis** | | | |
| Discussion | | |  |
| 17 | Summary of findings | Summarize the main interpretive findings of the translation and synthesis and compare them to existing literature | Discussion |
| 18 | Strengths, limitations, and reflexivity | Reflect on and describe the strengths and limitations of the synthesis: |  |
|  |  | ‐ Methodological aspects—for example, describe how the synthesis findings were influenced by the nature of the included studies and how the meta‐ethnography was conducted. | Discussion |
|  |  | ‐Reflexivity—for example, the impact of the research team on the synthesis findings | Discussion |
| 19 | Recommendations and conclusions | Describe the implications of the synthesis | Discussion |

**Supplementary Material Three**

**Table 4.** Dates for checking additional sources

| **Additional source checked** | **Date** |
| --- | --- |
| Reference lists of previous meta-syntheses | 16^th^ October 2020 |
| Reference lists of the included studies | 16^th^ October 2020  14^th^ November 2021 |
| Forward citation searching | 27^th^ October 2020  14^th^ November 2021 |
| Correspondence with all study authors regarding further publications | All were contacted between the 16^th^ and 27^th^ October 2020, with a further two contacted on 14^th^ November 2021 |
| Correspondence with authors for further clarification | Between the 26^th^ October 2020 (search 1) and 26^th^ November 2021 (updated search). |

**Supplementary Material Four**

**Table 5** below highlights the seven steps of conducting a meta-ethnography (Noblit & Hare, 1988). In practice, it is not unusual for steps to be iterative and non-linear, as well as overlapping with one another (France et al., 2019; Noblit & Hare, 1988).

**Table 5.** Meta-ethnography steps

| Step | Task |
| --- | --- |
| 1 | Formulating the research question  Identifying the need for a meta-ethnographic approach in an area of interest. |
| 2 | Deciding what is relevant  Defining the focus, systematic search, selection of studies, and quality assessment |
| 3 | Repeated reading of the studies, becoming familiar with and noting key concepts, themes, metaphors, and author interpretations.  Coding data verbatim into 1^st^ and 2^nd^ order constructs on NVivo  Extracting of key study characteristics |
| 4 | Deciding how the studies relate to/differ from one another. Examining relationships between studies and condensing themes into relevant categories. |
| 5 | Translation of studies into one another using reciprocal and/or refutational translations. Comparison and condensing of categories from each paper into all other papers, ensuring contextual meaning within and across studies is preserved to form translations which will give rise to 3^rd^ order constructs in step 6 |
| 6 | Synthesising translations using a line of argument synthesis to give new concepts or new understandings |
| 7 | Expressing the synthesis |

**Supplementary Material Five**

**Sensitivity Analyses**

There were no major differences identified between the second-order and first-order constructs. However, it should be noted that there was less depth within these first-order categories, compared with second-order categories, with fewer studies identified per category and indeed some studies did not include any first-order constructs (participant quotes).

**Table 6.** Step 4. First-order categories across studies incorporating author themes, subthemes, descriptions and interpretations (chronological order)

| **Category** | **Studies**^†^ |
| --- | --- |
| Mindfulness practice | 1, 2, 3, 9, 10, 11, 12, 13, 15, 16, 18, 20, 21 |
| Positive aspects of MBCT | 2, 3, 4, 5, 10, 11, 12, 14, 15, 16, 17, 18, 20, 21 |
| Awareness | 1, 2, 4, 5, 10, 11, 12, 14, 15, 16, 18, 19, 21 |
| Agency, choice, control | 1, 2, 3, 4, 5, 13, 14, 15, 16, 17, 19, 21 |
| Perspective | 1, 2, 3, 4, 5, 9, 10, 11, 12, 13, 14, 15, 18 |
| Relationships with self | 2, 5, 11, 12, 13, 14, 15, 16, 19, 20, 21 |
| Relationships with experience | 1, 2, 5, 10, 11, 12, 13, 14, 15, 16, 18, 19, 21 |
| Group processes | 1, 2, 3, 4, 5, 10, 11, 12, 13, 15, 16, 17, 18, 20, 21 |
| Relationships with others | 2, 4, 5, 11, 13, 14, 15, 17, 18, 19, 20, 21 |
| Acceptance | 1, 2, 3, 5, 10, 18, 19, 21 |
| Expectations | 1, 2, 3, 4, 5, 14, 15, 16, 17, 18 |
| Difficult aspects of MBCT | 2, 3, 4, 13, 15, 16, 20 |
| Differing changes and difficulties | 4, 11, 13, 14, 18 |
| Prior experiences | 1, 2, 5, 11, 14, 18 |
| Models of depression | 1, 5, 11, 14, 19 |

*Note.* ^†^Studies: 1=Mason & Hargreaves; 2=Ma; 3=Finucane & Mercer; 4=Smith et al.; 5=Allen et al.; 6=Cebolla & Barrachina; 7=Baillie et al.; 8=Hopkins & Kuyken; 9=Worsfold; 10=Ames et al.; 11=Bihari & Mullan; 12=Boggs et al.; 13=Lilja et al.; 14=Murphy & Lahtinen; 15=Di Toro; 16=Chesin et al.; 17=Racey et al.; 18=Williams et al.; 19=Tickell et al.; 20=Canby et al.; 21=Williams et al.

We also conducted sensitivity analyses based on the study characteristics. The purpose of this was to allow us to explore whether there were any noticeable differences between the overarching themes and second-order constructs, based on the country of origin (UK/Europe vs USA), method of MBCT delivery (face:face vs online), and depression status (full/partial remission vs current symptoms). There were some minor differences between themes within and across studies. These are displayed in **Table 7** below and are discussed throughout the results and discussion section of the paper.

**Table 7.** Sensitivity analyses based on study characteristics and organised by overarching themes and second-order constructs

| **Overarching theme** | **2nd-order constructs** | **Country** | | **MBCT delivery** | | **Depression status** | |
| --- | --- | --- | --- | --- | --- | --- | --- |
|  |  | UK/Europe  Total possible studies: (*N*=17) | USA  Total possible studies: (*N*=4) | Face:face  Total possible studies: (*N*=20) | Online  Total possible studies: (*N*=1) | Full/partial remission  Total possible studies: (*N*=15) | Current symptoms  Total possible studies: (*N*=6) |
| **Becoming skilled and taking action** | Practices | *N*=13  76% | *N*=2  50% | *N*=16  80% | *N*=1  100% | *N*=14  93% | *N*=3  50% |
|  | Intentions to Practice | 10  59% | 1  25% | 11  55% | 0 | 8  53% | 3  50% |
|  | 3-Minute Breathing Space (3MBS) | 3  18% | 1  25% | 4  20% | 0 | 1  0.07% | 3  50% |
|  | Awareness and Perspective | 15  88% | 2  50% | 16  80% | 1  100% | 13  87% | 4  67% |
|  | Agency and control around depression | 14  82% | 3  75% | 16  80% | 1  100% | 13  87% | 4  67% |
| **Acceptance** | Acceptance | 15  88% | 0 | 15  75% | 0 | 13  87% | 2  33% |
|  | Aliveness | 13  76% | 2  50% | 14  70% | 1  100% | 12  80% | 3  50% |
|  | Acceptance of depression | 3  18% | 0 | 3  15% | 0 | 3  20% | 0 |
|  | New understanding of depression | 6  35% | 0 | 6  30% | 0 | 6  40% | 0 |
|  | Permission around self-care | 13  76% | 2  50% | 14  70% | 1  100% | 13  87% | 2  33% |
|  | Group process: a safe and shared experience | 14  82% | 3  75% | 17  85% | 0 | 12  80% | 5  83% |
|  | Relationships with others | 11  65% | 3  75% | 13  65% | 1  100% | 11  73% | 3  50% |
| **Ambivalence and**  **Variability** | Group processes: difficulties | 6  35% | 4  100% | 9  45% | 1  100% | 5  33% | 5  83% |
|  | Confronting difficulty | 5  29% | 2  50% | 7  35% | 0 | 4  27% | 3  50% |
|  | Challenging but enjoyable | 4  24% | 1  25% | 5  25% | 0 | 2  13% | 3  50% |
|  | Differing changes and difficulties | 5  29% | 0 | 5  25% | 0 | 5  33% | 0 |
|  | Expectations | 10  59% | 3  75% | 12  60% | 1  100% | 8  53% | 5  83% |
|  | MBCT engagement | 13  76% | 3  75% | 15  75% | 0 | 11  73% | 5  83% |

*Note.* All studies delivered MBCT via groups, regardless of the method of delivery (face:face, online); proportions are calculated based on the number of studies reporting the specific sub-theme/second-order construct out of the total possible studies for that specific study characteristic; statistics are highlighted in yellow when there is >30% discrepancy between the proportion of studies reporting on the specific theme; study number 16 included participants in full/partial remission but who also expressed current suicidal ideation therefore this study was included as meeting criteria for current symptoms.

**Supplementary Material Six**

**Table 8.** Step 4. 2^nd^ order categories across studies incorporating author themes, subthemes, descriptions and interpretations

|  |  | | |  | | | | **Studies**^†^ | | | | | | | | | | | | | | | | |
| --- | --- | --- | --- | --- | --- | --- | --- | --- | --- | --- | --- | --- | --- | --- | --- | --- | --- | --- | --- | --- | --- | --- | --- | --- |
| **2^nd^ order constructs (categories)**  Sub-Categories | **1** | **2** | **3** | | **4** | **5** | **6** | | **7** | **8** | **9** | **10** | **11** | **12** | **13** | **14** | **15** | **16** | **17** | **18** | **19** | **20** | **21** | **Totals**^‡^ |
| **Mindfulness practice** | | | | | | | | | | | | | | | | | | | | | |  |  | **21** |
| Benefits |  |  |  | |  |  |  | |  |  |  |  |  |  |  |  |  |  |  |  |  |  |  |  |
| Difficulties (psychological, practical/logistical) |  |  |  | |  |  |  | |  |  |  |  |  |  |  |  |  |  |  |  |  |  |  |  |
| Styles (formal, informal, proactive/reactive use) |  |  |  | |  |  |  | |  |  |  |  |  |  |  |  |  |  |  |  |  |  |  |  |
| Motivations/intentions to continue |  |  |  | |  |  |  | |  |  |  |  |  |  |  |  |  |  |  |  |  |  |  |  |
| **Positive aspects of MBCT** | | | | | | | | | | | | | | | | | | | | | |  |  | **19** |
| Useful, helpful, enjoyable |  |  |  | |  |  |  | |  |  |  |  |  |  |  |  |  |  |  |  |  |  |  |  |
| Positive effects on mental health |  |  |  | |  |  |  | |  |  |  |  |  |  |  |  |  |  |  |  |  |  |  |  |
| Calmer, relaxed |  |  |  | |  |  |  | |  |  |  |  |  |  |  |  |  |  |  |  |  |  |  |  |
| Experiencing more positive emotions |  |  |  | |  |  |  | |  |  |  |  |  |  |  |  |  |  |  |  |  |  |  |  |
| Challenging but worth it |  |  |  | |  |  |  | |  |  |  |  |  |  |  |  |  |  |  |  |  |  |  |  |
| **Awareness** | | | | | | | | | | | | | | | | | | | | | |  |  | **18** |
| Experience (thoughts, emotions, sensations, reactions, and actions/behaviours) |  |  |  | |  |  |  | |  |  |  |  |  |  |  |  |  |  |  |  |  |  |  |  |
| Warning signals, coping strategies, and relapse risk |  |  |  | |  |  |  | |  |  |  |  |  |  |  |  |  |  |  |  |  |  |  |  |
| **Agency, choice, and control** | | | | | | | | | | | | | | | | | | | | | |  |  | **17** |
| Agency and choice over ADMs |  |  |  | |  |  |  | |  |  |  |  |  |  |  |  |  |  |  |  |  |  |  |  |
| Choice around thoughts, feelings, and reactions |  |  |  | |  |  |  | |  |  |  |  |  |  |  |  |  |  |  |  |  |  |  |  |
| Agency and control around ability to cope, depression, warning signs, ADMs, relapse |  |  |  | |  |  |  | |  |  |  |  |  |  |  |  |  |  |  |  |  |  |  |  |
| **Relationships with experience** | | | | | | | | | | | | | | | | | | | | | |  |  | **17** |
| Stopping/slowing, step back, space from experience |  |  |  | |  |  |  | |  |  |  |  |  |  |  |  |  |  |  |  |  |  |  |  |
| Reacting vs responding, relating more mindfully |  |  |  | |  |  |  | |  |  |  |  |  |  |  |  |  |  |  |  |  |  |  |  |
| Reduced rumination, depressive thinking styles |  |  |  | |  |  |  | |  |  |  |  |  |  |  |  |  |  |  |  |  |  |  |  |
| **Relationships with the self** | | | | | | | | | | | | | | | | | | | | | |  |  | **17** |
| Self-care, acknowledging needs and feelings |  |  |  | |  |  |  | |  |  |  |  |  |  |  |  |  |  |  |  |  |  |  |  |
| Struggle with self-care/prioritising self |  |  |  | |  |  |  | |  |  |  |  |  |  |  |  |  |  |  |  |  |  |  |  |
| Ongoing self-blame, guilt, shame |  |  |  | |  |  |  | |  |  |  |  |  |  |  |  |  |  |  |  |  |  |  |  |
| Emotion regulation |  |  |  | |  |  |  | |  |  |  |  |  |  |  |  |  |  |  |  |  |  |  |  |
| Confidence, feelings of empowerment, autonomy |  |  |  | |  |  |  | |  |  |  |  |  |  |  |  |  |  |  |  |  |  |  |  |
| Changing feelings towards depression identity, self-compassion, self-worth, self-criticism |  |  |  | |  |  |  | |  |  |  |  |  |  |  |  |  |  |  |  |  |  |  |  |
| **Group processes** | | | | | | | | | | | | | | | | | | | | | |  |  | **17** |
| Shared experience |  |  |  | |  |  |  | |  |  |  |  |  |  |  |  |  |  |  |  |  |  |  |  |
| Social experience |  |  |  | |  |  |  | |  |  |  |  |  |  |  |  |  |  |  |  |  |  |  |  |
| Safety, trust, validation (with group or instructor) |  |  |  | |  |  |  | |  |  |  |  |  |  |  |  |  |  |  |  |  |  |  |  |
| Difficulty with the group/lack of group experience |  |  |  | |  |  |  | |  |  |  |  |  |  |  |  |  |  |  |  |  |  |  |  |
| Group support |  |  |  | |  |  |  | |  |  |  |  |  |  |  |  |  |  |  |  |  |  |  |  |
| **Perspective** | | | | | | | | | | | | | | | | | | | | | |  |  | **17** |
| Thoughts, emotions, behaviour |  |  |  | |  |  |  | |  |  |  |  |  |  |  |  |  |  |  |  |  |  |  |  |
| Self-perspective |  |  |  | |  |  |  | |  |  |  |  |  |  |  |  |  |  |  |  |  |  |  |  |
| Present moment, appreciating life, more positive outlook |  |  |  | |  |  |  | |  |  |  |  |  |  |  |  |  |  |  |  |  |  |  |  |
| **Acceptance** | | | | | | | | | | | | | | | | | | | | | |  |  | **16** |
| Of experience (thoughts, emotions, pain/sensations) |  |  |  | |  |  |  | |  |  |  |  |  |  |  |  |  |  |  |  |  |  |  |  |
| Depression as an illness |  |  |  | |  |  |  | |  |  |  |  |  |  |  |  |  |  |  |  |  |  |  |  |
| ADMs |  |  |  | |  |  |  | |  |  |  |  |  |  |  |  |  |  |  |  |  |  |  |  |
| Self |  |  |  | |  |  |  | |  |  |  |  |  |  |  |  |  |  |  |  |  |  |  |  |
| Towards others/by others |  |  |  | |  |  |  | |  |  |  |  |  |  |  |  |  |  |  |  |  |  |  |  |
| Struggles with acceptance |  |  |  | |  |  |  | |  |  |  |  |  |  |  |  |  |  |  |  |  |  |  |  |
| **Relationships with others** | | | | | | | | | | | | | | | | | | | | | |  |  | **15** |
| Positive change: enhanced connections, intimacy, tolerance/patience, understanding others’ needs |  |  |  | |  |  |  | |  |  |  |  |  |  |  |  |  |  |  |  |  |  |  |  |
| Ongoing difficulties relating to others |  |  |  | |  |  |  | |  |  |  |  |  |  |  |  |  |  |  |  |  |  |  |  |
| No changes |  |  |  | |  |  |  | |  |  |  |  |  |  |  |  |  |  |  |  |  |  |  |  |
| Connection with something bigger |  |  |  | |  |  |  | |  |  |  |  |  |  |  |  |  |  |  |  |  |  |  |  |
| Relationship with the instructor |  |  |  | |  |  |  | |  |  |  |  |  |  |  |  |  |  |  |  |  |  |  |  |
| **Expectations** | | | | | | | | | | | | | | | | | | | | | |  |  | **13** |
| High expectations, expecting a cure |  |  |  | |  |  |  | |  |  |  |  |  |  |  |  |  |  |  |  |  |  |  |  |
| Disappointment |  |  |  | |  |  |  | |  |  |  |  |  |  |  |  |  |  |  |  |  |  |  |  |
| Open mind, no preconceptions |  |  |  | |  |  |  | |  |  |  |  |  |  |  |  |  |  |  |  |  |  |  |  |
| Impact of expectations on outcome |  |  |  | |  |  |  | |  |  |  |  |  |  |  |  |  |  |  |  |  |  |  |  |
| Wanted more support during/after MBCT |  |  |  | |  |  |  | |  |  |  |  |  |  |  |  |  |  |  |  |  |  |  |  |
| Concerns pre-MBCT |  |  |  | |  |  |  | |  |  |  |  |  |  |  |  |  |  |  |  |  |  |  |  |
| **Difficult aspects of MBCT** | | | | | | | | | | | | | | | | | | | | | |  |  | **10** |
| Practical/logistical barriers |  |  |  | |  |  |  | |  |  |  |  |  |  |  |  |  |  |  |  |  |  |  |  |
| Difficulty engaging in, or dislikes about, MBCT |  |  |  | |  |  |  | |  |  |  |  |  |  |  |  |  |  |  |  |  |  |  |  |
| Vulnerability, psychological and physical health barriers. Transient negative effects |  |  |  | |  |  |  | |  |  |  |  |  |  |  |  |  |  |  |  |  |  |  |  |
| **Variance of change** | | | | | | | | | | | | | | | | | | | | | |  |  | **6** |
| Lifelong change |  |  |  | |  |  |  | |  |  |  |  |  |  |  |  |  |  |  |  |  |  |  |  |
| Variable change |  |  |  | |  |  |  | |  |  |  |  |  |  |  |  |  |  |  |  |  |  |  |  |
| **Models of depression** | | | | | | | | | | | | | | | | | | | | | |  |  | **5** |
| Beliefs about causes of and treatment for depression |  |  |  | |  |  |  | |  |  |  |  |  |  |  |  |  |  |  |  |  |  |  |  |
| Different understanding/relationship with depression |  |  |  | |  |  |  | |  |  |  |  |  |  |  |  |  |  |  |  |  |  |  |  |
| **Prior experiences/descriptions of depression** | | | | | | | | | | | | | | | | | | | | | |  |  | **4** |
| Disconnection with, fear of upsetting others |  |  |  | |  |  |  | |  |  |  |  |  |  |  |  |  |  |  |  |  |  |  |  |
| Helplessness, perceived inability to cope |  |  |  | |  |  |  | |  |  |  |  |  |  |  |  |  |  |  |  |  |  |  |  |
| Descriptions of previous depression |  |  |  | |  |  |  | |  |  |  |  |  |  |  |  |  |  |  |  |  |  |  |  |

*Note.* ^†^Studies: 1=Mason & Hargreaves; 2=Ma; 3=Finucane & Mercer; 4=Smith et al.; 5=Allen et al.; 6=Cebolla & Barrachina; 7=Baillie et al.; 8=Hopkins & Kuyken; 9=Worsfold; 10=Ames et al.; 11=Bihari & Mullan; 12=Boggs et al.; 13=Lilja et al.; 14=Murphy & Lahtinen; 15=Di Toro; 16=Chesin et al.; 17=Racey et al.; 18=Williams et al.; 19=Tickell et al.; 20=Canby et al.; 21=Williams et al. ‡total number of studies within each category; ADMs=antidepressant medication

**Supplementary Material Seven**

**Reflexivity Statement**

This analysis was completed whilst the lead author was finalising their training as a Clinical Psychologist. KW has professional and personal experience with MBCT and related mindfulness-based approaches. KW has experience with teaching MBCT groups, holds a personal meditation practice, holds doctoral qualifications regarding MBCT, and has published scientific papers on MBCT and relevant mindfulness-based approaches. KW is aware that both their professional and personal interest in MBCT, and mindfulness-based meditation more generally, may impact on the analysis and results presented here. KW maintained this awareness throughout the process of conducting and analysing this study, and regularly reflected on this in a reflexive log and in discussions with the wider research team. SH, SL, and PT authors are academic and/or clinical practitioners (Clinical Psychologist: SH; Clinical Psychologist and Senior Lecturer: PT; Senior Lecturer: SL). MS (Trainee Clinical Psychologist) and MM (Assistant Psychologist) were involved in study screening, selection, and quality appraisals. PT and SH supervised the study. All authors acknowledge that their clinical and academic knowledge and experience may have an impact on the study results. However, whilst KW held arguably more knowledge and specific training in MBCT, other authors did not have this specific experience and knowledge around MBCT and, we believe, were therefore able to balance out the potential impacts on the credibility of the findings. Thus, regular discussions and reflections were held within the wider study team as a way to mitigate against this. All authors in the supervisory team allocated time to discussing their experience with MBCT and/or mindfulness, and any potential conflicts of interest around the topic at the start. All authors in the supervisory team set an agreement around being open, accepting, and respectful of views, whether similar or differing, throughout the analysis process.

**Supplementary Material Eight**

**Table 9.** Full text screening decisions

| **Article** | | **Decision** | **MBCT** | **Depression** | **Adults/ Adolescents** | **English written** | **Qualitative analysis** | **Comment** |
| --- | --- | --- | --- | --- | --- | --- | --- | --- |
| **Papers Included** | | | | | | | | |
| 1 | Allen, M., et al. (2009). | Yes | Yes | Yes | Yes | Yes | Yes |  |
| 2 | Ames, C. S., et al. (2014). | Yes | Yes | Yes | Yes | Yes | Yes |  |
| 3 | Baillie et al. (2012) | Yes | Yes | Yes* | Yes | Yes | Yes | *The sampling was focused on recruiting parents who had histories of recurrent depression and had participated in MBCT |
| 4 | Bihari, J. L. N. and E. G. Mullan (2014). | Yes | Yes | Yes | Yes | Yes | Yes |  |
| 5 | Boggs, J. M., et al. (2014). | Yes | Yes* | Yes | Yes | Yes | Yes | *Online MBCT delivery |
| 6 | Canby, N. K., et al. (2021) | Yes | Yes | Yes | Yes | Yes | Yes | Mixed methods. Only the qualitative data was analysed in this review |
| 7 | Cebolla i Marti and Barrachina (2009). | Yes | Yes | Yes | Yes | Yes | Yes |  |
| 8 | Chesin, M. S., et al. (2018). | Yes | Yes | Yes | Yes | Yes | Yes |  |
| 9 | Di Toro, B. M. (2017). | Yes* | Yes | Yes | Yes | Yes | Yes | *PhD thesis |
| 10 | Finucane, A. and S. W. Mercer (2006). | Yes | Yes | Yes | Yes | Yes | Yes |  |
| 11 | Hopkins, V. and W. Kuyken (2012). | Yes* | Yes | Yes | Yes | Yes | Yes | *This study specifically asked participants about their experiences of the MBCT reunions sessions (which take place after the course has finished). Following our inclusion criteria, this study was included because we wanted participants who have taken part in MBCT. |
| 12 | Lilja, J. L., et al. (2015). | Yes | Yes | Yes | Yes | Yes | Yes |  |
| 13 | Ma (2002) | Yes* | Yes | Yes | Yes | Yes | Yes | *PhD thesis |
| 14 | Mason, O. and I. Hargreaves (2001). | Yes | Yes | Yes | Yes | Yes | Yes |  |
| 15 | Murphy, H. and M. Lahtinen (2015). | Yes | Yes | Yes | Yes | Yes | Yes |  |
| 16 | Racey, D. N., et al. (2018). | Yes* | Yes | Yes | Yes | Yes | Yes | *This study was a feasibility and acceptability study with a TA qualitative analysis.  The study included young people’s, parents, and clinician views. Only the young peoples’ views were included. Clinician views were excluded as there was no data on whether clinicians had previous experiences with depression and they did not take part in MBCT. Parents’ views were excluded as although parents did have prior experiences with depression, the aim of the MBCT group for parents was to better support their children through depression and help to model principles of MBCT for their children. |
| 17 | Smith, A., et al. (2007) | Yes | Yes | Yes | Yes | Yes | Yes |  |
| 18 | Tickell (2020) | Yes | Yes | Yes | Yes | Yes | Yes |  |
| 19 | Williams, C. M., et al. (2018). | Yes | Yes | Yes | Yes | Yes | Yes |  |
| 20 | Williams, K., et al. (2021) | Yes | Yes | Yes | Yes | Yes | Yes |  |
| 21 | Worsfold, K. E. (2013). | Yes | Yes | Yes | Yes | Yes | Yes |  |
| **Papers Excluded** | | | | | | | | |
| 1 | Alsubaie, M., et al. (2018). | No | No* | Yes* | Yes | Yes | Yes | *Participants had a diagnosis of cardiovascular disease and a history of major depression or current mild depression symptoms. The main reason for exclusion, however, is **because the focus and themes of MBCT were adapted significantly** to include teaching relevant to cardiovascular disease (CVD) and **therefore not specific to depression and relapse prevention** (called MBCT-HELM (Heart and Living Mindfully)). More specifically, the **focus was moved away from depressive relapse and instead moved onto recognising bodily experiences associated with CVD.** There was also a shift from focusing on depressogenic thinking instead onto ways in which physical symptoms were interpreted.  **The intervention has changed and no longer focuses on both depression and relapse**; as outlined in our PROSPERO form: *“There may be some deviations to MBCT in its manualised form; such amendments are fine so long as the MBCT course does not deviate so much that it becomes MBSR or another MBI which does not contain content specific to depression and relapse.”* |
| 2 | Chesin, M. S., et al. (2015). | No | Yes* | Yes | Yes | Yes | No* | ***No qualitative analysis**, no themes present |
| 3 | Foulk, M. A., et al. (2014). | No | Yes | Yes* | Yes | Yes | No* | *Not all participants had experienced depression  *This was a feasibility/accessibility study and **did not include any qualitative analysis** and instead only presented case studies. |
| 4 | Ewais, T., et al. (2020) | No | Yes* | Yes* | Yes | Yes | Yes | *The authors say that MBCT was adapted for participants with both IBD and depression. They provide baseline demographics for IBD statistics but do not give demographics related to depression (e.g., rating scale, previous/no of episodes). The authors adapted the content of MBCT to focus on flares, stress, and shorter practices etc. Adolescents. MBCT but they have both depression and IBD |
| 5 | Garriz, M., et al. (2020). | No* | Yes | Not all* | Yes | Yes | Yes | *The qualitative study took place within the context of a mixed-method study. Total sample=269 participants, of which **only 23% had a diagnosis of depression**. Others in the total sample had diagnoses of anxiety (14%), personality disorder (5%), and/or adjustment difficulties (42%). Other diagnoses were not listed. Findings were shared across all participants therefore it is **difficult to know which themes arose in those participants with diagnoses of depression**, specifically, making it hard to compare and draw conclusions re themes across all relevant, included studies.  **As only 14/269 participants took part in the qualitative aspect of the study, it is difficult to know whether any of these 14 participants had a diagnosis of depression**. |
| 6 | Hortynska, K., et al. (2016). | No | Yes | Unclear* | Yes | Yes | Yes | *Some participants had experienced depression but the authors had not categorised their sample by diagnostic categories therefore the sample also included many other 'diagnoses', **meaning it was difficult to identify experiences specific to people with depression**.  Other presenting problems included anxiety, PTSD, bipolar, body dysmorphia, early trauma, worries, rumination, panic attacks, work stress, OCD, chronic pain, self-criticism, complex bereavement, self-blame, social anxiety, health anxiety, generalized anxiety, health problems, suicidal ideation and intrusive thoughts |
| 7 | Huijbers, M. J., et al. (2020) | No | Yes | Yes | Yes | Yes | Yes* | Mixed methods. Only the qualitative data was analysed in this review  The qualitative analysis focused on participants’ experiences of tapering/discontinuing antidepressants. Themes were related to their experience of tapering, not to MBCT content or experience of the benefits or challenges of the course itself. |
| 8 | Hunter-Jones, J. J., et al. (2019). | No | No* | Yes* | Yes | Yes | Yes | *Adapted MBCT into a program called UPLIFT (Using Practice and Learning to Increase Favourable Thoughts) which when looking at the session content, **it is different to standardised MBCT** (e.g., the UPLIFT program in this study was developed for people with epilepsy and CF, does not introduce meditation until session 4, includes more CBT exercises than standardised MBCT, relaxation, and has less emphasis on relapse prevention).  *Participants had diagnoses of comorbid HIV/AIDS and depression (although this is not the reason for exclusion) |
| 9 | Hunter-Jones, J. J., et al. (2021). | No | No* | No* | Yes | Yes | Yes | *Adapted MBCT into a program called UPLIFT (Using Practice and Learning to Increase Favourable Thoughts) which when looking at the session content, **it is different to standardised MBCT.**  *Not specific to participants experiencing depression. |
| 10 | Kristofersson, G. K. (2013). | No | No* | No* | Yes | Yes | Yes | ***Not MBCT.** MBSR (Mindfulness-based Stress Reduction)  *Participants **did not have diagnoses of depression** but instead diagnoses of substance misuse and traumatic brain injury |
| 11 | Langdon et al., 2011 | No | Yes | Unclear* | Yes | Yes | Yes | *Participants had experienced depression, anxiety, or chronic health problems (e.g., heart disease). One MBCT course was run only for physical illness and the other was for depression & anxiety and **it is not possible to identify/isolate which themes are taken from which course/which depressed sample** |
| 12 | Lockman, S. C. (2015). | No* | No* | Yes* | Yes | Yes | Yes | Thesis  ***Not MBCT** as participants were only required to have had a basic mindfulness practice for the last 8 weeks. |
| 13 | Lomas, T., et al. (2015). | No | No* | No* | Yes | Yes | Yes | ***Not MBCT,** general meditation experience  ***Not specific to depression** |
| 14 | Malpass, A., et al. (2015). | No | Yes* | No* | Yes | Yes | Yes | *Primary diagnoses were asthma & Chronic Obstructive Pulmonary Disease (COPD), then depression and/or anxiety as secondary diagnoses. Therefore, **experiences of depression and/or anxiety were a secondary focus of the study**.  Further, **there is no data on diagnoses of depression/anxiety and so it is hard to tell what proportion of the sample had this, and therefore whether the themes relate to experiences of depression (the focus of our review).** Overall, the themes of this study were primarily focused with experiences of COPD and asthma.  *MBCT was delivered but the session content was slightly adapted to fit with learning acceptance towards living with asthma and COPD |
| 15 | Nangia & Sharma, 2012 | No* | Yes* | Yes | Yes | Yes | No | *Although the method states TA, there is no further detail given and only titles of themes given in the results section, with no supporting data (quotes, descriptions, or interpretations). Therefore, this has been excluded due to there being **no qualitative analysis**  *Some minor adaptations to the length of MBCT (e.g., ranging between 8-12 sessions), which incorporated one-to-one assessment sessions either side of the 8 week group. |
| 16 | Rycroft-Malone et al., (2019) | No* | Yes | Not all* | Yes | Yes | Yes | *The sample **included only 16 clients out of a sample of 91** (which included service managers, commissioners, and MBCT teachers) making it hard to draw out useable data re clients’ themes |
| 17 | Schuling, R. et al., (2021) | No* | No* | Yes | Yes | Yes | Yes | *The authors investigated Mindfulness-based Compassionate Living (MBCL) which is a follow-on course for MBCT. The course content is adapted from MBCT and includes a more explicit focus on self-compassion. |
| 18 | Smith, A. (2004). | No* | Yes* | No* | Yes | Yes | Yes | *The author combined 6 groups - **3 MBSR** for anxiety/chronic pain and 3 MBCT groups for recurrent depression. So the themes emerged based on these 6 combined groups meaning that **half the sample didn't have depression and didn’t take part in an MBCT group specific to depression.** |
| 19 | Smith, A. (2006). | No* | Yes | Yes | Yes | Yes | Yes | This book chapter reported exactly the same data that was later published in Smith (2007) paper which is included above in the synthesis. Therefore, it is excluded **here to avoid duplication.** |
| 20 | Smith, E. (2012). | No* | Yes | Yes | Yes | Yes | Yes | *This is the earlier dissertation/thesis from Smith (2015) which **was later published and is included in our synthesis above.** |
| 21 | Smith, E. L., et al. (2015). | No | Yes* | Not all* | Yes | Yes | Yes | *This study involved clients and their partners. **Clients had a history of depression but their partners did not.** Themes were focused on the experiences of taking part in **MBCT as a couple**. The themes here **were developed based on both participant and partners**, so not two separate groups and therefore **themes could not be isolated.**  Only slight adaptations made to MBCT and so it still focused on relapse prevention |
| 22 | Ter Avest, M. J., et al. (2019). | No* | Yes | Yes | Yes | Yes | No* | ***No qualitative analysis**. |
| 23 | Weaver, 2015 | No* | Yes | Yes | Yes | Yes | Yes | *Upon closer examination, we found that **this thesis was the precursor to the later published paper** by Tickell et al. (2020; included above in the included studies; both studies have the same sample sizes and characteristics) therefore it was treated as a duplication here. This was also clarified with the original study authors. It was decided within the wider research team to only include the themes by Tickell et al. (2020) as this included additional follow-up data, a more comprehensive analysis process, and had been through the peer-review process. *Doctoral thesis |

*Note*. TA=Thematic Analysis

**Supplementary Material Nine**

**Table 10.** CASP quality results by study

| **Study**^†^ | **1. Aims** | **2.**  **Qual. methods** | **3. Design** | **4.**  **Recruitment strategy** | **5.**  **Data collection** | **6. Participant researcher relationship** | **7. Ethical issues** | **8. Rigorous analysis** | **9. Statement of findings** | **10. Value** | **Comment** |
| --- | --- | --- | --- | --- | --- | --- | --- | --- | --- | --- | --- |
| 1 | ✓ | ✓ | ✓ | ? | ✓ | ✓ | 🗶 | ✓ | ✓ | ? | 4 –more detail needed regarding participant drop out/retention  7 – No details for ethical approval or consent  10 – more detail needed |
| 2* | ✓ | ✓ | ✓ | ✓ | ✓ | 🗶 | ✓ | ✓ | ? | ? | 6 – no detail  9 – could benefit from a summary around key findings  10 – limited mention of key contributions/ new avenues for research  *unpublished PhD thesis |
| 3 | ✓ | ✓ | ? | ✓ | ✓ | ? | ✓ | ✓ | ✓ | ✓ | 3 – justification of choice of qualitative method is not clear  6 – brief statement in the method but more detail could be given |
| 4 | ✓ | ✓ | ✓ | ✓ | ? | ? | ? | ✓ | ✓ | ✓ | 5 – could benefit from a topic/interview guide  6 – some detail given, but could be more explicit  7 – the authors mention informed consent but do not mention NHS/local ethical approval  The authors only illustrate three participant case studies in detail |
| 5 | ✓ | ✓ | ✓ | ✓ | ✓* | ? | ✓* | ✓ | ✓ | ✓ | 5 - Interview schedule not given  6 - Although author experience and potential biases are listed, there is no real analysis regarding reflexivity and the role of the researchers  7 – more detail needed regarding informed consent and confidentiality |
| 6 | 🗶 | ✓ | ? | 🗶 | ✓ | 🗶 | 🗶 | 🗶 | ? | 🗶 | 1 – study aims not clear  3 –it is hard to tell whether the research design is appropriate  4 – no specific detail about recruitment (e.g., selection of participants, details around attrition: one part mentions 31 responses, another mentions 32)  6 – no reflexivity mentioned  7 – no mention of ethics  8 – brief mention of content analysis.  9 – needs more discussion around credibility of findings  10 – unclear what the implications of the findings are  *study is suspected to be a service evaluation but unclear |
| 7 | ✓ | ✓ | ✓ | ✓ | ✓ | ✓ | ? | ✓ | ✓ | ✓ | 7 – more detail needed for consent & confidentiality |
| 8 | ✓ | ✓ | ✓ | ✓ | ✓ | ✓ | ✓ | ✓ | ✓ | ✓ | 5 – more detail could be given around the interview topic guide (if there was one) |
| 9 | ✓ | ✓ | ? | ✓ | ✓ | 🗶 | ? | ? | ? | ✓ | 3 – unclear on analysis  6 – no detail given  7 – the author states that participants gave informed written consent but there are no details regarding ethical approval  8 – analysis method not clear  9 - no details on credibility of findings |
| 10 | ✓ | ✓ | ? | ? | ? | ? | ✓ | 🗶 | ✓ | ✓ | 3 – no justification regarding the choice of qualitative methods  4 – more detail could be given  5 – more detail needed around the interview schedule  6 – acknowledgement that the researcher has both a personal and professional interest in MBCT, but no expansion around the potential impact of this  8 – no in-depth discussion around the analysis. No acknowledgement of reflexivity. Limited quotes from participants |
| 11 | ✓ | ✓ | ✓ | ✓ | ✓ | ✓ | ✓ | ✓ | ✓ | ✓ |  |
| 12 | ✓ | ✓ | ? | ? | ✓ | ? | ✓ | ✓ | ✓ | ✓* | 3 – no justification for the choice of qualitative methods  4 - no interviews with participants who didn’t complete the full course despite there initially being 100 participants in the study. No explanation why they dropped out.  6 – brief acknowledgement in the discussion regarding the potential impact of the relationship between researchers and participants. Nothing in relation to the design or analysis process, however.  10 – mostly this is considered, but some more detail needed |
| 13 | ✓ | ✓ | ✓ | ✓ | ✓ | ✓ | ✓ | ✓ | ✓ | ✓ |  |
| 14 | ✓ | ✓ | ✓ | ✓ | ✓ | ✓ | ✓ | ✓ | ✓ | ✓ |  |
| 15 | ✓ | ✓ | 🗶 | ✓ | ✓ | ? | ✓ | ✓ | ✓ | ✓ | 3 – no sufficient detail around justification of the qualitative design, specifically the choice of thematic analysis.  6 – brief statement around the potential for the halo effect whereby participants wanted to answer positively for the researcher.  *unpublished doctoral thesis |
| 16 | ✓ | ✓ | ? | ✓ | ✓ | 🗶 | ✓ | ✓ | ✓ | ✓ | 3 - some justification of the method but more detail about the analysis method needed  6 – a brief statement is given regarding the authors’ beliefs about the programme efficacy but the link between that and outcome is not made explicit. |
| 17 | ✓ | ✓ | 🗶 | ✓ | ✓ | 🗶 | ✓ | ? | ? | ✓ | 3 – justification of choice of qualitative method is unclear  6 – no consideration  8 – they say they developed a topic guide but we can’t see it  9 – could benefit from more detail regarding credibility of findings (e.g., triangulation, multiple analysts)  *service evaluation |
| 18 | ✓ | ✓ | ✓ | ✓ | ✓ | ?* | ✓ | ✓ | ✓ | ✓ | 6 – authors feedback their themes to study participants and disclosed their experience with MBCT; no specific statement of the potential for bias |
| 19 | ✓ | ✓ | ? | ✓ | ✓ | ✓ | ✓ | ✓ | ✓ | ✓ | 3 - justification of choice of TA is unclear  Good reflection on researcher relationship, topic guide & booklets although some may be leading questions. |
| 20 | **✓** | **✓** | **✓** | **?** | **✓** | **?** | **✓** | **?** | **✓** | **✓** | 4 - limited information about recruitment and if people chose not to take part.  6 - minimal information about the potential researcher bias although interviews were conducted by study personal to reduce demand characteristics.  8 - limited information on data analysis and how data was selected. |
| 21 | **✓** | **✓** | **✓** | **✓** | **✓** | **✓** | **✓** | **✓** | **✓** | **✓** |  |

*Note.* ^*^additional information given or needed here; Individual item ratings are rated either ✓=“Yes”, 🗶=“No, or ?=“Can’t tell”; ^†^Studies: 1=Mason & Hargreaves; 2=Ma; 3=Finucane & Mercer; 4=Smith et al.; 5=Allen et al.; 6=Cebolla & Barrachina; 7=Baillie et al.; 8=Hopkins & Kuyken; 9=Worsfold; 10=Ames et al.; 11=Bihari & Mullan; 12=Boggs et al.; 13=Lilja et al.; 14=Murphy & Lahtinen; 15=Di Toro; 16=Chesin et al.; 17=Racey et al.; 18=Williams et al.; 19=Tickell et al.; 20=Canby et al.; 21=Williams et al. The quality appraisal for paper number 21 was not carried out by the lead author as they were the lead author on this paper, but instead carried out by two of the co-authors (MM; MS).

**Supplementary Material Ten**

**Prior Experiences with Depression**

Five studies explicitly described participants’ prior experiences with depression, largely reflecting negative feelings towards the self and interpersonal difficulties. **Table 10** below includes details and an example quote.

**Table 11.** Second-order construct and example quote

| **2^nd^-order constructs** | **Translation of 2^nd^-order construct** | **2^nd^-order example quote (studies*)** | **Studies**^†^ |
| --- | --- | --- | --- |
| Prior experiences of depression | Feelings of helplessness, withdrawal from others, and efforts to put on a “front”. Struggles with “problematic thinking”. | “Prior to MBCT the majority of participants stated that they had viewed depression as an opaque process over which they felt helpless” (5) | 5, 7, 11, 14, 19 |

*Note.* ^†^Studies: 5=Allen et al.; 7=Baillie et al.; 11=Bihari & Mullan; 14=Murphy & Lahtinen; 19=Tickell et al.

Specifically, participants described feelings of helplessness, guilt, and inadequacy around experiences of depression, and named “problematic thinking” and “thought overload” (Mason & Hargreaves, 2001) alongside struggles to "extricate" themselves from downward trajectories (Murphy & Lahtinen, 2015). Some commented on difficulties with interpersonal relationships and increased levels of reactivity to others (“criticising others…getting caught up in distressing, repetitive rows and arguments”; Bihari & Mullan, 2014). Some described “withdrawing and feeling isolated from others” (Bihari & Mullan, 2014), and having “learned to mask their feelings” (Allen et al., 2009) from others to give the perception that they could cope.
